# A Review on Carbon Dots: Synthesis, Characterization and Its Application in Optical Sensor for Environmental Monitoring

**DOI:** 10.3390/nano12142365

**Published:** 2022-07-11

**Authors:** Nur Alia Sheh Omar, Yap Wing Fen, Ramli Irmawati, Hazwani Suhaila Hashim, Nur Syahira Md Ramdzan, Nurul Illya Muhamad Fauzi

**Affiliations:** 1 Faculty of Science, Universiti Putra Malaysia (UPM), Serdang 43400, Selangor, Malaysia; nuralia.upm@gmail.com (N.A.S.O.); irmawati@upm.edu.my (R.I.); hazwanisuhaila@gmail.com (H.S.H.); nursyahira.upm@gmail.com (N.S.M.R.); 2 Institute of Nanoscience and Nanotechnology, Universiti Putra Malaysia (UPM), Serdang 43400, Selangor, Malaysia; illyafauzi97@gmail.com

**Keywords:** carbon dots, green synthesis, chemical synthesis, optical sensor, environmental pollution

## Abstract

The development of carbon dots (CDs), either using green or chemical precursors, has inevitably led to their wide range application, from bioimaging to optoelectronic devices. The reported precursors and properties of these CDs have opened new opportunities for the future development of high-quality CDs and applications. Green precursors were classified into fruits, vegetables, flowers, leaves, seeds, stem, crop residues, fungi/bacteria species, and waste products, while the chemical precursors were classified into acid reagents and non-acid reagents. This paper quickly reviews ten years of the synthesis of CDs using green and chemical precursors. The application of CDs as sensing materials in optical sensor techniques for environmental monitoring, including the detection of heavy metal ions, phenol, pesticides, and nitroaromatic explosives, was also discussed in this review. This profound review will offer knowledge for the upcoming community of researchers interested in synthesizing high-quality CDs for various applications.

## 1. Introduction

Fluorescent carbon dots (CDs), also known as carbon quantum dots (CQDs) or carbon nanodots (CNs), have drawn a great deal of attention in recent years, owing to their high photostability, excellent water solubility, tuneable fluorescence and optical properties, low toxicity, good biocompatibility, and environmental friendliness [1]. To date, a variety of precursors have been utilized to prepare CDs through “bottom-up” or “top-down” approaches [2]. Among these approaches, hydrothermal/carbonization treatment is frequently applied for the preparation CDs because of the outstanding advantages, such as high yield, simple manipulation, easy control, uniform products, lower air pollution, low energy consumption and so on [3]. Despite these advantages, CDs cannot be produced without the presence of starting materials, also known as precursors. In this regard, the development of green and chemical synthesis methods for producing high fluorescent CDs has gathered the focus of researchers. The green synthesis methods by means of green precursors of synthesis involves the usage of inexpensive or recycled materials, while the chemical synthesis methods involve toxic chemical reagents or organic solvents as precursors. CDs synthesized using these methods usually contain a certain amount of oxygen, hydrogen, and nitrogen. With all these options to prepare CDs under certain experimental conditions, it is no surprise that differential fluorescence properties of CDs were easily acquired. In addition, surface passivation (generally involves functional groups such as amine groups and hydroxyl groups) and the heteroatom doping (n-type doping: nitrogen, phosphorus, sulfur, and chlorine or p-type doping: boron) approach is another factor that paves the way to efficiently improve the fluorescent properties, quantum yield, and other physicochemical properties of CDs [4]. Such intriguing properties have triggered new capabilities in the field of sensing and bioimaging.

To date, many review articles on the preparation of CDs, heteroatom doped CDs, and their applications have been published [5,6,7,8]. However, there is no comprehensive review exploring various green and chemical precursors as the carbon source in CDs and their application in environmental monitoring. The purpose of this review is, therefore, to update and organize the green and chemical precursors used in CD preparation. The final section will give a concise overview of their application in the optical sensing of heavy metal ions, phenol, pesticides, and nitroaromatic explosives.

## 2. CDs Synthesized from Green Precursors

Carbon dots (CDs) can be synthesized using two precursors, either from green or chemical sources. As of now, researchers focus more on simple, low-cost, and greenway synthesis for the large-scale production of high-quality CDs. Numerous green sources, such as fruits, vegetables, flowers, leaves, seeds, stems, crop residues, fungi/bacteria species and waste products, have been used as a carbon source for the preparation of CDs. In this section, all green sources used for the synthesis of CDs are briefly arranged and compared.

### 2.1. Fruits

Orange juice and watermelon peels were among the very first precursors used as a carbon source to synthesize CDs. The studies reveal the as-prepared CDs have different fluorescence properties, due to the presence of different chemical groups in the raw materials and synthesis method. The synthesized CDs from orange juice showed the quantum yield of 26% at an emission of 441 nm, whereas a 7.1% quantum yield was demonstrated by watermelon peel-derived CDs at an emission of 490–580 nm [9,10]. In 2014, Du et al. reported renewable wastes of sugar cane bagasse as a new precursor for fluorescent CDs [11]. It, thus, demonstrated that such bagasse-derived CDs could function as highly effective fluorescent sensing probes for labeling and imaging in biomedical applications.

Lemon peels, prunus avium extract, cornstalk, corn bract, dried lemon peels, pulp-free lemon juice, citrus lemon peels, citrus sinensis peels, etc. are another precursor that have been used in the development of CDs, as shown in Table 1 [12,13,14,15,16,17,18,19,20,21,22,23,24,25,26,27,28,29,30,31,32,33,34,35,36,37,38,39,40,41]. Among these precursors, acidic fruits, such as lemon peels, lemon juice, and citrus sinensis, were frequently chosen. This is because the juice extract is rich in sucrose, glucose, fructose, citric acid, and ascorbic acid, while the peels are mainly composed of proteins, fibers, and less of oils and antioxidants. Consequently, CDs from juice extract exhibit higher fluorescence properties than peels, due to the high acid and sugar contents that provide a considerable amount of carbon and hydrogen elements. A comparison study of the different precursors used in hydrothermal synthesis was then carried out to understand the role of citric acid [30]. It was found that lemon juice has higher photoluminescence (PL) emission than ripe lemon juice and orange juice. This result is because lemon juices have higher concentrations of citric acid than orange juices. Moreover, the decrease in PL emission of ripe lemon juice was due to a significant decrease in the grade of constituents and destroyed their surface structure.

**Figure 1:**
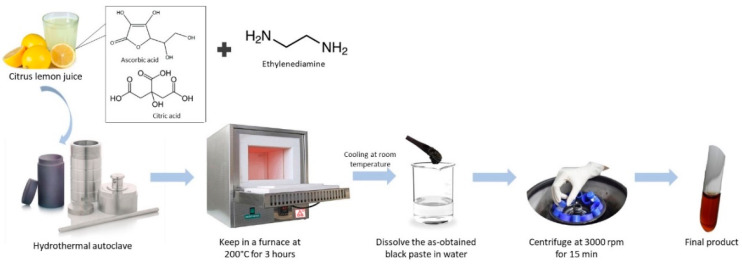
Schematic of hydrothermal reaction of CDs from citrus lemon juice and ethylenediamine.

Instead of just relying on carbon sources to produce high quantum yields of CDs, it has been found that by passivating CDs with amine-containing molecules, such as ethylenediamine, one can increase the quantum yield and selectively sensing analytes. Figure 1 shows the hydrothermal reaction of CDs from citrus lemon juice in the presence of ethylenediamine to produce nitrogen-doped CDs. The results showed that the prepared nitrogen-doped CDs had the quantum yield of 31% under bright blue emission [28]. However, there are limits to these blue emissive CDs, especially in the field of bioimaging because of strong tissue autofluorescence at low wavelength emissions. Herein, Ding et al. (2017) have heated an ethanol solution of pulp-free lemon juice to produce very high red-luminescent CDs, which hold the promise for in vitro and in vivo bio-imaging [17].

At present, Bael patra fruit-derived CDs turned out to be an excellent carbon source without the need of chemical additives [39]. Typically, three different components of Bael patra fruit, i.e., hard shell, edible pulp, and a mixture of pulp and gum were reported for the successful synthesis of emissive CDs, and it was found that CDs derived from the hard shell had the highest quantum yield of 59.39%. This is because the synthesized CDs via the carbonization approach resulted in more production of carbon content and micropores over its surface. These micropores can provide a higher ratio of surface-active sites for the adsorption of toxins from wastewater samples, thus significantly improving the sensing performance.

### 2.2. Vegetables

The exploration of new carbon sources that possess abundant reserve, green, simple and high quality CDs has drawn tremendous attention in the area of kitchen waste, such as vegetables. Various green and non-green vegetables, such as celery leaves, sweet pepper, lemon grass, tomato, carrot, rose-heart radish, turmeric, cinnamon, red chili, black pepper, hongcaitai, cauliflower, kelp, tomato, crown daisy leaves, cabbage, cherry tomatoes, scallion leaves, and red beet, have been reported, as shown in Table 2 [42,43,44,45,46,47,48,49,50,51,52,53,54,55,56,57,58]. Both green and non-green vegetables have many properties and their own advantages. For instance, the family of green vegetables, such as celery leaves, lemon grass, hongcaitai, kelp, cabbage, crown daisy leaf, and scallion, often contain many organic compounds, such as organic acids, amides, amino acids, proteins, saccharides, carbohydrates, chlorophyll, etc., which can bring good physical and chemical properties to CDs. Meanwhile, non-green vegetables, such as tomato, red chili, turmeric, black pepper, cinnamon, and red beet, are plants rich in various bioactive compounds of lycopene, capsaicin, curcumin, piperine, and cinnamaldehyde, respectively, which enable their application in the biomedical field. It was reported that bioactive compounds will partially remain inside or at the surface of the CDs after the hydrothermal process, leading to different photoluminescent and biomedical properties. Vasimalai et al. (2018) have demonstrated the uses of cinnamon, red chili, turmeric, and black pepper as CD precursors for biomedical applications [48]. They found that black pepper CDs have the highest quantum yield of 43.6% due to the various functional groups present in the sample, namely O–H, C–H, C–O–N, C=O, C–O, and C–N vibrational stretching peaks. All in all, the CDs prepared using celery leaves have contributed to the highest quantum yield of 53%. It was discovered that celery leaves contain abundant folic acid with affluent -COOH and the addition of L-glutathione as N, S- dopant has enriched their surface groups, which are beneficial for improving the quantum yield of CDs [42].

### 2.3. Flowers

Flowers such as *Selenicereus grandifloras*, water hyacinth, *Osmanthus fragrans*, rose flowers, and *Tagetes erecta* have also shown promise as carbon precursors, which were subsequently used to bind pesticides and metal ions. From Table 3, we can observe that there is an increase in quantum yield from 3.8% in 2019 to 63.7% in 2021. This significant improvement was attained by the presence of surface-active organic groups of C–N, –NH, and –OH, as shown in the FTIR analyses [59,60,61,62,63].

In another study, Shekarbeygi et al. (2020) evaluated the effect of rose pigments (blue, red, and yellow) and the effect of their extraction methods (aqueous and alcohol) on optical properties of the synthesized CDs [62]. The results indicated that the quantum yields obtained for all the CDs were not affected by the rose pigments and extraction methods, as the yields were almost the same. However, such rose pigments and extraction methods affected the CDs’ thermal stability and emission wavelengths. In the case of thermal stability, the fluorescence intensities were reduced in both aqueous and alcoholic CDs, with an increase in temperature ranging from 17 to 57 °C, yet aqueous CDs showed a higher decreasing rate than alcoholic CDs. As for the emission wavelengths, the alcohol extract had a larger wavelength than the aqueous extract, which may be due to the change in dielectric constant of solvent and the presence of more phenolic groups in the alcoholic extract. Thus, this work showed that CDs prepared with alcoholic extract with yellow petals has higher stability and a longer emission wavelength with better quantum yield than the others.

### 2.4. Leaves, Seeds, and Stems

In this section, we focused on the parts of plant-derived CDs, namely leaves (such as *Ocimum sanctum* leaves, bamboo leaves, gingko leaves, *Gynostemma* leaves, betel leaves, *Calotropis procera* leaves, *Elettaria cardamomum* leaves, Cornus walteri leaves, tea leaves, Kentucky bluegrass), seeds (such as *Acacia concinna* seeds, fennel seeds, *Pearl millet* seeds), and stem (lotus root), as presented in Table 4 [64,65,66,67,68,69,70,71,72,73,74,75,76,77,78]. Most of them have excellent healing properties, such as anti-inflammatory, antioxidant, antimicrobial, and antidote properties. Due to those health benefits, the synthesized CDs could serve simultaneously in the fluorescent sensing of metal ions and as contrast agents for live cells [64,65,66]. Nonetheless, more efforts are still needed to explore the effect of reaction temperature, time, and pH, despite the remarkable use of carbon precursors. The work of Dager et al. (2019) witnessed complete carbonization of fennel seeds (hydrocarbon converted to the graphitic structure) when the reaction temperature increased to the highest temperature at 500 °C for 3 h [70]. It was also found that by heating the sample longer (for 5 h) did not result in any significant change in either PL or crystallinity. Furthermore, when the synthesized CDs were dispersed in water at different pHs ranging from 3 to 13, the emission intensity gradually increased from acidic to basic media and eventually decreased when the reaction pH was higher than 9.

**Figure 2:**
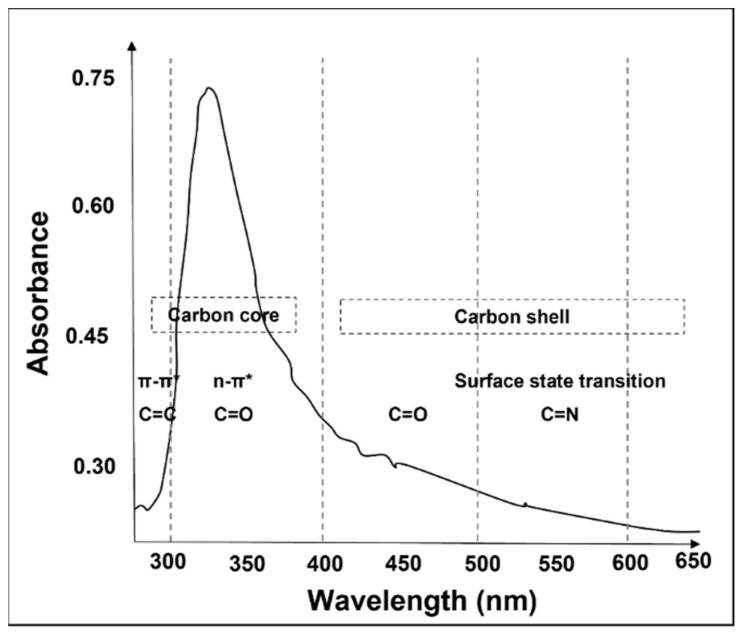
UV–Vis spectra of CDs from *Calotropis procera* leaves through hydrothermal method at 200 °C for 4 h. Reproduced with copyright permission of Elsevier [73].

Leaves are recognized as the most excellent carbon precursors by reason of exhibiting higher quantum yields than seeds and stem. Most interestingly, the *Calotropis procera* leaf-derived CDs provided an excellent quantum yield (71.95%) without any toxic agents or surface passivation chemicals [73]. It is noteworthy that an excellent quantum yield resulted from the functional groups (OH, N–H, C=O, and C=N bond) derived from the carbon precursors itself. Furthermore, the synthesized CDs have a strong peak around 320 nm in the UV–Vis spectra of CDs (Figure 2), implying that it was spawned by the n → π* transition of C=O bonds over the surface of CDs.

### 2.5. Crop Residues

Crop residues, such as sago waste, palm kernel shell, and wheat straw, are another potential carbon source used in the green preparation of CDs (Table 5). In the year of 2014, Tan et al. successfully demonstrated the conversion of sago waste into fluorescent CDs via thermal pyrolysis without any surface passivation [79]. The heating temperature of pyrolysis was found to alter the degree of carbonization of bulk sago waste into carbonaceous residues. It was observed that heating treatments of lower than 400 °C can lead to incomplete carbonization, while higher temperatures can cause severe decomposition of the organic structures in sago waste into ashes, which leads to the loss of the fluorescing property. The optimum temperature of carbonization was found to be 400 °C. However, the synthesized CDs may suffer from low quantum yields due to the absence of solvent.

Solvents, especially solvents with high boiling points, play an important role in providing better carbonization rates for the formation of CDs. Of these, controlling the reaction conditions by adding solvent is of particular importance. Thus, to date, diethylene glycol [80], ultrapure water and ethanol [81], and deionized water [82] have been used as solvents to improve carbonization efficiency. The results showed that the reaction of diethylene glycol (DEG) on palm kernel shell-derived CDs had the highest quantum yield of 44%. This advancement is due to the higher boiling point of DEG than the others, thus allowing a more complete reaction for the formation of CDs at higher temperatures.

### 2.6. Fungi/Bacteria Species

The conversion of fungi/bacteria species into a value-added product such as carbonaceous nanomaterials has contributed to the green and sustainable improvement. Algal blooms [83], yogurt [84], enokitake mushroom [85], microalgae biochar [86], mushroom [87], agarose waste [88], and *Shewanella oneidensis* [89] have been previously chosen as a carbon resource (Table 6). A study conducted by Pacquiao et al. (2018) demonstrated the increment of quantum yield of 11% to 39% upon passivation with tetraethylenepentamine in the presence of 5% *v/v* sulfuric acid [85]. The XPS spectrum revealed the presence of small signals of nitrogen and sulfur at 399 eV (N_1s_) and 168 eV (S_2p_), respectively, thus confirming the successful heteroatoms doping on CDs and sulfuric acid as a reagent. Furthermore, the effect of temperatures (180, 200, and 250 °C) and reaction times (4, 6, and 8 h) on the quantum yield was evaluated, yielding the optimum temperature and reaction time at 250 °C for 4 h. The research, thus, concluded that higher temperatures and shorter reaction times can produce higher quantum yields. Even so, CDs prepared from agarose waste-derived CDs without using other passivation chemicals exhibited the highest quantum yield of 62% [88]. This quantum efficiency can be attributed to the presence of different functional groups (–C=O, –OH, and N–H) on the surface of CDs, in addition to being related to the surface defects and the particle size of CDs.

### 2.7. Waste Products

**Figure 3:**
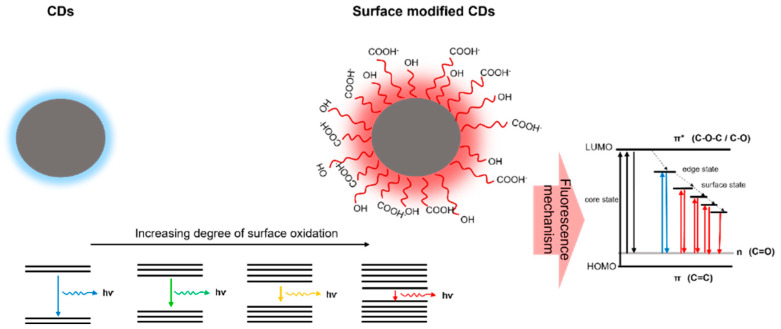
Scheme illustration of tunable PL of CDs with different degrees of oxidation.

There are ongoing efforts to improve the photoluminescent properties while reducing the production cost and protecting the environment. Of the different plant and fungi sources, the reuse of waste (Table 7), such as frying oil [90], biocrude oil [91], polystyrene [92], tea powders [93], expired milk [94], coal powder [95], papers [96], polyolefin [97], polypropylene plastic waste [98], bike soot [99], etc. [100,101,102,103,104,105,106,107,108,109,110,111], has become one of the hottest scientific research topics nowadays for the development of CDs. Among these materials, plastic waste and heavy oil products offer a promising precursor, as they have produced high quantum yields of more than 60%. In the case of plastic waste, cup-derived CDs have displayed the highest quantum yield value of 65% at 310 nm compared to bottles (64%) and polybags (62%) [108]. The high quantum yield of CDs is highly dependent on the carbonyl groups in CDs, revealing the presence of NH_2_. Furthermore, all the three prepared CDs displayed a slight red shift in the emission peak, with respect to the excitation wavelength. These behavioral variations were mainly due to the presence of epoxy and hydroxyl functional groups in the CDs prepared from the plastic waste, which resulted in creating new energy levels between n-π* gaps. Due to this reason, the energy gaps of the CDs between the lowest unoccupied molecular orbital (LUMO) and the highest occupied molecular orbital (HOMO) became reduced with the increasing degree of surface oxidation, thereby contributing to the enhanced fluorescence property (Figure 3).

Recently, the precursors with higher molecular weight, higher heteroatoms and higher aromatic structures were shown to be more conducive to the production of CDs. Ma et al. (2021) observed that CDs synthesized with asphalt as the precursor have the highest fluorescence quantum yield compared to heavy oil, light deasphalted oil (LDAO), and heavy deasphalted oil (HDAO) [110]. The reason is that the CDs from asphalt had the highest aromatic carbon ratio and the lowest naphthenic carbon ratio and alkyl carbon ratio. In addition, the asphalt had higher molecular weight, more oxygen, nitrogen and sulfur contents, and higher carbon/hydrogen atomic ratio than the other precursors. This result was further confirmed by the presence of the doublet γ peak and (002) peak in the X-ray diffraction pattern of asphalt, which indicated that the asphalt had a great quantity of graphite-like ordered structures.

## 3. CDs Synthesized from Chemical Precursors

Although many natural precursors have been used in the preparation of low-cost CDs, chemical precursors are still being explored to this day to produce high quantum yields of CDs. This section reviews two types of reaction chemical precursors used in CD preparation, namely acid and non-acid reagents, which were briefly summarized in Table 8 and Table 9.

### 3.1. Acid Reagents

Since 2012, carbon sources based on acid reagents, such as citric acid, phosphoric acid, acetic acid, folic acid, ascorbic acid, sodium citrate, ascorbic acid, glutamic acid, malonic acid, maleic anhydride, boric acid, pyrogallic acid, phthalic acid, 3-aminobenzeneboronic acid, succinic acid, p-aminosalicylic acid, diethylenetriamine-pentacetate acid, maleic anhydride, DL-thioctic acid, sulfamic acid, tartaric acid, 2-aminoterephthalic acid, trans-aconitic acid, dehydroabietic acid, dithiosalicylic acid, etc., have been intensively employed in the production of CDs [112,113,114,115,116,117,118,119,120,121,122,123,124,125,126,127,128,129,130,131,132,133,134,135,136,137,138,139,140,141,142,143,144,145,146,147,148,149,150,151,152,153,154,155,156,157,158,159,160,161,162,163,164,165,166,167,168,169,170,171,172,173,174,175,176,177,178,179,180,181,182,183,184,185,186,187,188,189,190,191,192,193,194,195,196,197].

Dong et al. (2013) first compared the fluorescence properties of the following three types of CDs: (i) O-CDs synthesized from citric acid only, (ii) N-CDs synthesized from citric acid and glycine, and (iii) N,S-CDs synthesized from citric acid and L-cysteine [113]. It was observed that the doping of nitrogen into O-CDs can introduce a new kind of surface state (labelled as the N-state). Electrons trapped by the new formed surface states are able to facilitate a high yield of radiative recombination. Although the quantum yield of N-CDs was found to be higher than that of O-CDs, the fluorescence spectra were still broad and excitation-dependent. In such cases, the introduction of sulfur atoms into CDs could lead to more significant enhancement, offering higher yields and excitation-independent emission. The introduced sulfur atoms seem to be able to eliminate the O-states and enhance the N-state, meaning that the original surface states are almost neglected in the N,S-CDs. However, when the authors tuned the ratio of L-cysteine into citric acid, from 1 g:2 g into 0.125 g:2 g, the quantum yield of N,S-CDs decreased from 73% to 37% and the emission wavelength was dependent on excitation and yielded a red-shift from 415 to 540 nm under the excitation wavelength of 375 to 480 nm. Interestingly, when the excitation wavelength was set lower than 375 nm, the emission wavelength was excitation independent. Although the aforementioned CDs were highly enhanced by nitrogen and sulfur co-doping, the chemistry involved therein is extremely challenging due to the involvement of oxygen, let alone the interference from defects. Therefore, until single nitrogen doping-sources, such as urea, ethylenediamine, PEG diamine, melamine, etc., have been widely reported, as shown in Table 8.

On the other hand, citric acid as the carbon source was commonly used in the chemical synthesis of CDs. Citric acid has considerable advantages over other acid reagents, such as being relatively cheap, more sour, less harmful to the environment, and readily available in large commercial quantities. Consequently, two highest fluorescence quantum yields were attributed to the reflux reaction of citric acid and diethylenetriamine [133] and hydrothermal reaction of citric acid and ethylenediamine [176] at 82.40% and 85.69%, respectively. As expected, ethylenediamine as N-doping agents plays a major role in improving the fluorescence properties in CDs. By way of example, Wang et al. (2015) have reported the influence of three different polyethylenic amine molecules, i.e., ethylenediamine (EDA), diethylenetriamine (DETA), and tetraethylenepentamine (TEPA) with a combination of citric acid on photoluminescence performance [129]. The results showed that the CDs-EDA has the highest PL quantum yield at 69.3%, followed by CDs-DETA (68%) and CDs-TEPA (33.4%). The results imply that the increasing presence of cyclic imines (C=N) with the enhancement of conjugated π-domains in CDs imparts superior PL efficiency.

**Figure 4:**
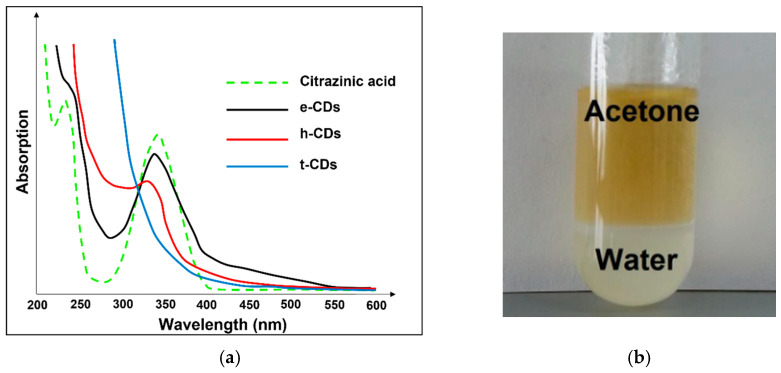
(**a**) Absorption spectra of three CD species and pure citrazinic acid. Adapted with permission from ref. [130]. Copyright 2017 American Chemical Society; (**b**) the salt-induced phase separation using acetone. Reproduced with copyright permission of Elsevier [137].

There is another study that reported the fabrication of CDs from different nitrogen sources, such as ethylenediamine (e), hexamethylenetetramine (h), and triethanolamine (t) [130]. From the X-ray photoelectron spectroscopy analyses, the ratios of carbon to nitrogen in these CDs were determined, namely, 83:17 for e-CDs, 86.14 for h-CDs, and 96:4 for t-CDs. Because of the high nitrogen content in e-CDs, the quantum yield was found to be highest at 53%. The amount of nitrogen can be typically correlated with a high photoluminescence quantum yield and confirms similar features in the absorption spectra (a shoulder at 234 nm and a broad peak at 340 nm) between e-CDs and pure citrazinic acid, the basic unit from the presumed class of fluorophores. However, at wavelengths longer than 400 nm, pure citrazinic acid has no absorption characteristic, unlike e, h and t-CDs, as shown in Figure 4a. The presence of broad absorption in this energy is commonly assigned to surface states related to the functional surface groups in CDs, which form low-energy sub-band gaps within the n−π* band gap.

Furthermore, Chang et al. (2017) reported that the phosphoric acid (H_3_PO_4_) or sucrose solution alone could not produce a significant fluorescence emission under the excitation of 423 nm [137]. However, when H_3_PO_4_ and sucrose solutions were mixed and incubated in the oven, the fluorescence light was emitted at the excitation wavelength of 423 nm. It is thought that the mild heating of H_3_PO_4_ has the power to hydrolyze sugar into simple carbon-rich molecules. Of note, to deactivate the carbonization process, the authors then added sodium hydroxide into CDs, but this appeared to cause a high salt content that was not favourable to many applications. Hence, acetone with a 1:1 volume-to-volume ratio was used to overcome this problem, resulting in two phases, as shown in Figure 4b. The highly ionic salt species remained in the water mother-liquor, while the CDs that were less ionic were partitioned into the acetone phase. The removal of CDs from acetone can be easily achieved via evaporation, since acetone is quite volatile. Based on this, the fluorescence signal of CDs was found to be increased by approximately 10%.

Recently, other carbon sources derived from amino acids, namely arginine, lysine, histidine, cysteine, and methionine have been reported [168,193,194,195]. This is because of their several advantages, for instance, non-toxic, biocompatible, and eco-friendly nature. Most importantly, each of the amino acids have different numbers of -COOH, -NH, and -SH functional groups, which can effectively improve the optical and chemical properties of CDs. Upon mixing these amino acids with citric acid via the microwave method, N and/or S-doped CDs were successfully synthesized in a very short time for the first time, which only took 1–4 min [168]. The reaction mechanism was proposed to include the following two steps: (i) reaction of carboxylic acid of citric acid with the -NH and –SH groups of amino acids via condensation polymerization and then the esterification of citric acid with amino acids, (ii) carbonization of these polymer clusters during irradiation by total dehydration to generate N and/or S doped CDs under microwave exposure. The research concluded that only cysteine-CDs were found to possess the highest quantum yield of 89.5% upon the N and S atom containing CDs.

The highest quantum yield of 93% was achieved when sodium citrate dihydrate and urea were used as precursors [179]. Sodium citrate dihydrate plays an important role as a self-assembly trigger for a carbon-based structure, due to the intermolecular H-bonding, while urea acts as nitrogen-doping precursors, which both are responsible for the increase in quantum yield. In another study, instead of using common synthesis methods such as oxidation, combustion, and hydrothermal, Ji et al. (2021) established another method, which is simpler and timesaving. Briefly, 0.34 g of L-cysteine, 0.15 g of urea, and 8.5 g of diphosphorus pentoxide were mixed, followed by the addition of 6 mL of water with a rapid stirring process [193]. These CDs were then embedded in in polyvinyl alcohol (PVA) gel substrate to form a smooth and high fluorescent CD/PVA film for further use in hexavalent chromium detection.

**Table 8:** Summary of the synthesis of CDs from acid reagents.

Precursor		Technique	Properties			Year	Reference
Carbon Source	Passivation/Solvent		Particle Size	Fluorescence	Quantum Yield		
Citric acid	Terbium (III) nitrate pentahydrate	Carbonization	TEM- 3 nm	λ_em_- 450 nm λ_ex_- 320 nm	-	2012	[112]
Citric acid monohydrate	L-cysteine	Hydrothermal treatment	HRTEM- 7 nm	λ_em_- 415 nm λ_ex_- 345 nm	73%	2013	[113]
	-		-	λ_em_- 435 nm λ_ex_- 345 nm	5.3%		
	Glycine		-	λ_em_- 415 nm λ_ex_- 345 nm	16.9%		
Citric acid	Urea	Microwave	TEM- 4 to 6 nm	λ_em_- 460 nm λ_ex_- 360 nm	0.13%	2013	[114]
Ethyleneglycol bis-(2-aminoethyl ether)- N,N,N′,N′-tetraacetic acid	Tris(hydroxymethyl)aminomethane	Thermal carbonization	TEM- 5 nm	λ_em_- 425 nm λ_ex_- 310 nm	28%	2014	[115]
Citric acid	PEG-diamine	Solid-phase	TEM- 1.7 nm	λ_em_- 435 nm λ_ex_- 360 nm	31%	2014	[116]
Citric acid	Poly(ethylenimine)	Pyrolysis	HRTEM- 3.5–4.5 nm	-	42.5%	2014	[117]
Folic acid	Ethylene glycol and nanopure water	Hydrothermal	TEM- 4.5 nm	λ_em_- 470 nm λ_ex_- 395 nm	15.7%	2014	[118]
Poly(ethylene glycol) and ascorbic acid	Distilled water	Microwave	TEM- 2.3 nm	λ_em_- 450 nm λ_ex_- 373 nm	-	2014	[119]
Citric acid	Ethylenediamine	Microwave-assisted pyrolysis	TEM- 3 nm	λ_em_- 455 nm λ_ex_- 280 nm	-	2015	[120]
Citric acid	L-Tyrosine methyl ester hydrochloride	Hydrothermal	TEM- 3.7 nm	λ_em_- 433 nm λ_ex_- 348 nm	3.8%	2015	[121]
Citric acid	Ethylenediamine and double distilled water	Hydrothermal	-	-	75.0%	2015	[122]
Citric acid	1-Aminopropyl-3-methy-imidazolium bromide	Pyrolysis	HRTEM- 0.6–1.6 nm	λ_em_- 440 nm λ_ex_- 380 nm	2.03–27.66%	2015	[123]
Glacial acetic acid	N-Acetyl-L-cysteine, diphosphorus pentoxide and distilled deionized water	Simple mixing	TEM- 2.51–3.44 nm	λ_em_- 480 nm λ_ex_- 300 nm	4.65%	2015	[124]
Citric acid	L-cysteine, urea and ultrapure water	Microwave	TEM- 1.1 nm	λ_em_- 450 nm λ_ex_- 353 nm	25.2%	2015	[125]
Citric acid	Dithiooxamide and distilled water	Microwave-assisted hydrothermal	STEM- 2 nm	λ_em_- 448 nm λ_ex_- 360 nm	17.6%	2015	[126]
Citric acid monohydrate	Ammonia and double distilled water	Hydrothermal	TEM- 3.7 nm	λ_em_- 442 nm λ_ex_- 350 nm	40.5%	2015	[127]
Sodium citrate	Urea and ultrapure water	Electrochemical carbonization	TEM- 2.4 nm	λ_em_- 433 nm λ_ex_- 351 nm	11.9%	2015	[128]
Citric acid anhydrous	Ethelendiamine	Condensation carbonization	TEM- 3.9 nm	λ_em_- 445 nm λ_ex_- 365 nm	69.3%	2015	[129]
	Diethylenetriamine		TEM- 3.7 nm		68%		
	Tetraethylenepentamine		TEM- 4.1 nm		33.4%		
Citric acid anhydrous	Ethylenediamine and deionized water	Hydrothermal	-	λ_em_- 440 nm λ_ex_- 320 nm	53%	2016	[130]
	Hexamethylenetetramine and deionized water			λ_em_- 420 nm λ_ex_- 320 nm	17%		
	Triethanol-amine and deionized water			λ_em_- 420 nm λ_ex_- 320 nm	7%		
Citric acid	Ethylenediamine and deionized water	Hydrothermal	TEM- 5 to 7 nm	λ_em_- 443 nm λ_ex_- 365 nm	-	2016	[131]
Citric acid	Branched polyethylenimine	Condensation	DLS- 1.9 nm	λ_em_- 450 nm λ_ex_- 350 nm	-	2016	[132]
Citric acid	Diethylenetriamine	Reflux treatment	TEM- 5 -7 nm	-	82.40%	2016	[133]
Ascorbic acid and valine	Ethanol and distilled water	Hydrothermal	TEM- 4 nm	λ_em_- 430 nm λ_ex_- 352 nm	4.8%	2016	[134]
L-glutamic acid	Silica gel powders and water	Microwave	TEM- 1.64 nm	λ_em_- 450 nm λ_ex_- 370 nm	41.2%	2016	[135]
Malonic acid	Urea and ultrapure water	Hydrothermal	TEM- 2.5 nm	λ_em_- 397 nm λ_ex_- 320 nm	12.6%	2017	[136]
Sucrose and phosphoric acid	Sodium hydroxide	Carbonization	SEM- 10 nm	λ_em_- 524 nm λ_ex_- 423 nm	-	2017	[137]
Citric acid	Sodium phosphate	Solid-phase	TEM- 1.7 nm	λ_em_- 435 nm λ_ex_- 360 nm	-	2017	[138]
Citric acid	Ethylenediamine and ultrapure water	Hydrothermal	TEM- <10 nm	λ_em_- 431 nm λ_ex_- 337 nm	32.25%	2017	[139]
Citric acid monohydrate	Thiourea	Microwave solid-phase pyrolysis	TEM- 2 nm	λ_em_- 436 nm λ_ex_- 358 nm	23.6%	2017	[140]
Citric acid	Silk	Hydrothermal	TEM- 5.6 nm	λ_em_- 425 nm λ_ex_- 360 nm	61.1%	2017	[141]
Citric acid	Melamine	Hydrothermal	TEM- 1.8 nm	λ_em_- 422 nm λ_ex_- 320 nm	8.11%	2017	[142]
Citric acid	Tartaric acid, ethanediamine and oleic acid	Solvothermal	TEM- 2.66 nm	λ_em_- 460 nm λ_ex_- 360 nm	42.2%	2017	[143]
Citric acid and urea	Water	Solvothermal	TEM- 1.7 nm	λ_em_- 448–638 nm λ_ex_- 375 nm	-	2017	[144]
	Glycerol		TEM- 2.8 nm				
	Dimethylformamide		TEM- 4.5 nm				
Maleic anhy- dride and tetraethylenepentamine	Sulfuric acid and deionized water	Pyrolysis	TEM- 20 nm DLS- 8 nm	λ_em_- 450 nm λ_ex_- 360 nm	21%	2017	[145]
D-(+)-maltose monohydrate, boric acid and thiocarbamide	-	Hydrothermal	TEM- 2.0 nm	λ_em_- 415 nm λ_ex_- 326 nm	8.9%	2017	[146]
Pyrogallic acid	N-N-dumethylformamide	Solvothermal	TEM- 11.9 nm	λ_em_- 520 nm λ_ex_- 360–450 nm	16.8%	2018	[147]
L-histidine and citric acid	Ethylene glycol	Polyol microwave	TEM- 19 nm	λ_em_- 430–511 nm λ_ex_- 350 nm	-	2018	[148]
Phthalic acid and triethylenediamine hexahydrate	Deionized water	Microwave	TEM- 2–6 nm	λ_em_- 520–542 nm λ_ex_- 360–440 nm	16.1%	2018	[149]
Citric acid	Urea	Solvothermal	TEM- 1.87 nm	λ_em_- 590 nm λ_ex_- 540 nm	43%	2018	[150]
Citric acid	Lysine and ultrapure water	Hydrothermal	TEM- 10 nm	-	-	2018	[151]
Citric acid monohydrate	-	Thermal treatment	TEM- 3.5 nm	λ_em_- 450 nm λ_ex_- 360 nm	3.54%	2018	[152]
Citric acid	Ammonium thiocyanate and deionized water	Microwave-assisted	HRTEM- 30 nm	λ_em_- 490 nm λ_ex_- 410 nm	-	2018	[153]
Folic acid and p-phenylenediamine	Sodium hydroxide	Hydrothermal	TEM- 2 nm	λ_em_- 505 nm λ_ex_- 420 nm	8.4%	2018	[154]
3-Aminobenzeneboronic acid	Deionized water	Hydrothermal	TEM- 3 nm	λ_em_- 504 nm λ_ex_- 400 nm	-	2018	[155]
Succinic acid	Deionized water and glycerol	Hydrothermal	TEM- 2.3 nm	λ_em_- 410 nm λ_ex_- 280 nm	11%	2018	[156]
			TEM- 4.6 nm	λ_em_- 525 nm λ_ex_- 480 nm	7%		
Phosphoric acid	Ethylenediamine	Simple heating (180 °C, 2 h)	TEM- 3.2 nm	λ_em_- 430 nm λ_ex_- 340 nm	5.17%	2018	[157]
		Simple heating (280 °C, 2 h)	TEM- 6.4 nm	λ_em_- 413 nm λ_ex_- 340 nm	21.8%		
Phosphoric acid and ethanolamine	Water	Microwave irradiation	TEM- 3.4 nm	λ_em_- 417 nm λ_ex_- 340 nm	20.52%	2018	[158]
P-aminosalicylic acid	Ethyleneglycol dimethacrylate and double distilled water	Hydrothermal	TEM- 3 nm AFM- 1.6 nm DLS- 11.7 nm	λ_em_- 520 nm λ_ex_- 390 nm	27.2%	2018	[159]
Sodium citrate	Urea and dimethylformamide	Solvothermal	TEM- 3.52 nm	λ_em_- 446 nm λ_ex_- 370 nm	67%	2018	[160]
Citric acid monohydrate	3-(Aminopro- pyl)triethoxysilane (APTES)	Thermal decomposition	TEM- 5–15 nm	λ_em_- 416 and 480 nm	-	2019	[161]
Citric acid	Urea and deionized water	Hydrothermal	-	-	-	2019	[162]
	Glycine and deionized water		-				
	Citric acid, deionized water, ethylene glycol, N,N’- bis(2-aminoethyl)-1,3-propanediamine		HRTEM- 5–6 nm				
Citric acid and urea	N,N-dimethylformamide	Solvothermal	-	λ_em_- 450, 550, 630 nm λ_ex_- 400–450 nm	-	2019	[163]
	N,N-dimethylformamide, NaOH and HCl		TEM- 3.7 nm	λ_em_- 630 nm λ_ex_- 550 nm	-		
	N,N-dimethylformamide, NaOH and water		TEM- 2.1 nm	-	-		
Procaine hydrochloride and citric acid	Double distilled water and ethylenediamine	Hydrothermal	TEM- 3.3 nm	λ_em_- 440 nm λ_ex_- 360 nm	47.1%	2019	[164]
Anhydrous citric acid	N-(β-aminoethyl)-γ-aminopropyl-methyldimethoxysilane	Hydrothermal	TEM- 2.22 nm	λ_em_- 460 nm λ_ex_- 370 nm	51.8%	2019	[165]
Citric acid	Thiourea and deionized water	Microwave-assisted pyrolysis	TEM- 3.3 nm	-	-	2019	[166]
Sodium citrate and aminopyrazine	Ultrapure water	Hydrothermal	TEM- 2.38 nm	λ_em_- 389 nm λ_ex_- 310 nm	11.8%	2019	[167]
Citric acid	Deionized water and arginine	Microwave	TEM- 11 ± 4 nm	λ_em_- 330 nm λ_ex_- 430 nm	3.9 ± 0.4%	2019	[168]
	Deionized water and lysine		TEM- 17 ± 2 nm	λ_em_- 330 nm λ_ex_- 430 nm	4.2 ± 1.9%		
	Deionized water and histidine		TEM- 6 ± 5 nm	λ_em_- 330 nm λ_ex_- 433 nm	2.8 ± 0.2%		
	Deionized water and cysteine		TEM- 10 ± 7 nm	λ_em_- 330 nm λ_ex_- 420 nm	89.5 ± 2.3%		
	Deionized water and methionine		TEM- 9 ± 5 nm	λ_em_- 330 nm λ_ex_- 407 nm	2.5 ± 0.6%		
Citric acid and phenylalanine	Ultrapure water	Hydrothermal	TEM- 2–3 nm	λ_em_- 330 nm λ_ex_- 310 nm	-	2020	[169]
Polyacrylamide and citric acid	Ultrapure water	Hydrothermal	TEM- 4.1 nm	λ_em_- 330 nm λ_ex_- 310 nm	12.6%	2020	[170]
Citric acid and urea	-	Infrared carbonization	TEM- 5–10 nm	λ_em_- 475 nm λ_ex_- 360 nm	22.2%	2020	[171]
Citric acid and urea	-	Hydrothermal (180 °C, 20 min)	TEM- 2–7 nm	-	46% 26% 4%	2020	[172]
		(230 °C, 20 min)		λ_em_- 394, 440, 523 nm λ_ex_- 350 nm	23% 35% 36%		
Citric acid	Melamine and formaldehyde	Hydrothermal	TEM- 3.7 nm	λ_em_- 425 nm λ_ex_- 350 nm	63.7%	2020	[173]
Citric acid monohydrate	Urea	Microwave irradiation	TEM- 6 nm	λ_em_- 536 and 532 nm λ_ex_- 350 nm	-	2020	[174]
Citric acid	Phenylalanine	Hydrothermal	TEM- 11.9 nm	λ_em_- 413 nm λ_ex_- 350 nm	65%	2020	[175]
Citric acid monohydrate	Ethylenediamine	Hydrothermal	TEM- 5–10 nm	λ_em_- blue λ_ex_- 305–395 nm	85.69%	2020	[176]
Citric acid	Ethylenediamine and ultrapure water	Hydrothermal	TEM- 5 nm	λ_em_- 444 nm λ_ex_- 360 nm	-	2020	[177]
Ascorbic acid	Urea and deionized water	Microwave irradiation	TEM- 2 nm	λ_em_- 415 nm λ_ex_- 340 nm	7%	2020	[178]
Sodium citrate dihydrate	Urea and deionized water	Thermal pyrolysis	TEM- 2.75 nm	λ_em_- 525 nm λ_ex_- 400 nm	93%	2020	[179]
Citric acid and 3-aminobenzeneboronic	Dimethylformamide	Hydrothermal	HRTEM- 3.4 nm	-	-	2020	[180]
Diethylenetriamine- pentacetate acid	Ultrapure water	Carbonization	HRTEM- 2.85 nm	-	-	2020	[181]
Maleic anhydride and triethylenetetramine	Deionized water and nitric acid	Pyrolysis	TEM- 5.9 nm	λ_em_- 400 nm λ_ex_- 320 nm	6.3%	2021	[182]
DL-thioctic acid	Dimethylformamide, trisodium citrate dihydrate, sodium hydroxide, double deionized water	Hydrothermal	HRTEM- 2.52 nm	λ_em_- 438 nm λ_ex_- 340 nm	-	2021	[183]
Citric acid and sulfamic acid	Polyethyleneimine	Two-step hydrothermal	TEM- 5.1 nm	λ_em_- 460 nm λ_ex_- 355 nm	29.1%	2021	[184]
Tartaric acid	Urea	Solid-phase thermal	TEM- 4.13 nm	λ_em_- 537 nm λ_ex_- 460 nm	10.5%	2021	[185]
2-aminoterephthalic acid and polyethylene glycol	Orthophosphoric acid	Microwave-assisted pyrolysis	TEM- 3–10 nm	λ_em_- 470 nm λ_ex_- 410 nm	67%	2021	[186]
Citric acid	Ultrapure water and ethylenediamine	Hydrothermal	TEM- 3.1 nm	λ_em_- 445 nm λ_ex_- 356 nm	-	2021	[187]
Citric acid	Ethylenediamine and water	Microwave	TEM- 2.3 nm	λ_em_- 450 nm λ_ex_- 360 nm	-	2021	[188]
Trans-aconitic acid	Diethylenetriamine and distilled water	Hydrothermal	HRTEM- 2–8 nm	λ_em_- 435 nm λ_ex_- 345 nm	81%	2021	[189]
Dehydroabietic acid	Ethanolamine	Hydrothermal	TEM- 3.2 nm	λ_em_- 433 nm λ_ex_- 365 nm	10%	2021	[190]
Citric acid	L-glutamine	Hydrothermal	TEM- 3.5 nm	λ_em_- 450 nm λ_ex_- 360 nm	-	2021	[191]
	D-glutamine		TEM- 3–4 nm				
Dithiosalicylic acid	Acetic acid and o-phenylenediamine	Solvothermal	TEM- 4.5 nm	λ_em_- 620 nm λ_ex_- 560 nm	4.05%	2021	[192]
	Acetic acid and m-phenylenediamine		TEM- 4.0 nm	λ_em_- 560 nm λ_ex_- 460 nm	20.77%		
	Acetic acid and p-phenylenediamine		TEM- 3.5 nm	λ_em_- 478 nm λ_ex_- 460 nm	1.76%		
L-cysteine and urea	Diphosphorus pentoxide and water	One-pot synthesis	TEM- 4.5 nm	λ_em_- 445 nm λ_ex_- 362 nm	17%	2021	[193]
Methyl cellulose and L-cysteine	Ethylenediamine	Hydrothermal	TEM-19 nm	λ_em_- 370 nm λ_ex_- 330 nm	12.3%	2021	[194]
Ce (NO_3_)_3_·6H_2_O and L-histidine	Sodium hydroxide and deionized water	One-pot hydrothermal	SEM- 46 nm	-	-	2021	[195]
Polyethylenimine and citric acid	Hot water	Microwave-assisted	STEM- 12 nm	λ_em_- 442 nm λ_ex_- 354 nm	54%	2022	[196]
Citric acid and urea	Ultrapure water	Solvothermal	HRTEM- 3.18 nm	λ_em_- 470 nm λ_ex_- 330 nm	20.1%	2022	[197]
	20 mL dimethylformamide		HRTEM- 3.25 nm	λ_em_- 500 nm λ_ex_- 330 nm	22.1%		
	10 mL dimethylformamide and ethanol		HRTEM- 3.47 nm	λ_em_- 539 nm λ_ex_- 330 nm	21.9%		
	10 mL dimethylformamide and acetic acid		HRTEM- 3.68 nm	λ_em_- 595 nm λ_ex_- 330 nm	24.2%		

### 3.2. Non-Acid Reagents

**Figure 5:**
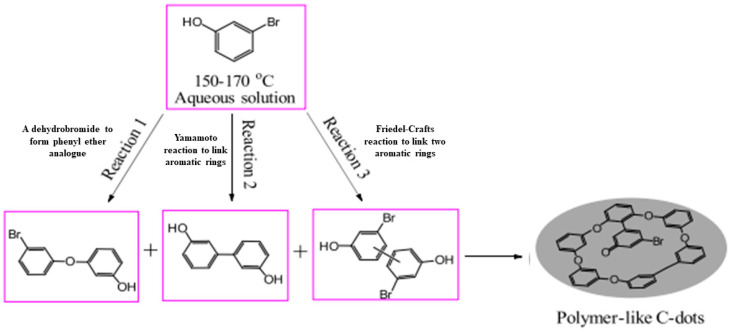
Proposed possible mechanisms for the formation CDs using 3–bromophenol. Reproduced with copyright permission of Elsevier [206].

Carbon sources based on non-acid reagents, namely graphite oxide [198], poly(ethylene glycol) [199,200], 3-(3,4-dihydroxyphenyl)-L-alanine [201], ethanolamine [202], polyimide [203], polyethyleneglycol bis(3-aminopropyl) [204], ethanol [205], 3-bromophenol [206], azidoimidizole [207], activated carbon [208], glucose [209], N-methylethanolammonium thioglycolate [209], chitosan [210], chlorophyll [211], microcrystalline cellulose [212], o-phenylenediamine [213], carbon paper [214], papain [215], dextrose solution [216], etc. [217,218,219,220,221,222,223,224,225,226,227,228,229,230], have also been reviewed, as shown in Table 9. 3-Bromophenol is the first insecticide adopted to be a potential precursor, thus suggesting that carbon sources are not limited in typical chemical reagents with high purity or low toxicity [206]. In this study, the effect of mixed solvent of ethanol and water ratio (*v*/*v*) such as 1:9, 3:7, 5:5, 7:3 and 9:1 and the effect of temperatures such as 150, 160, 170 °C were evaluated. The results indicate that both much thicker organic solvents and much higher carbonized temperatures are in favour of the formation of C-dots featuring mono-dispersion, high yield, and smaller size. The proposed mechanism behind the fabrication of insecticide-based CDs was verified by the occurrence of aromatic C-Br and Br anion, Csp2 C-C peak and Csp3 C-C peak found on the XPS spectrum. The three possible main classes of the reaction that happened among the 3-bromophenol molecules are given in Figure 5.

Efforts to develop and improve the fluorescence of the CDs have been made toward polymer compounds, including synthetic and natural polymers. Thanks to the toxicity of synthetic polymers, natural polymers extracted from plants have been used as a carbon source with the characteristics of being environment-friendly, non-toxic, low price, good biocompatibility, and stable chemical properties. For this reason, Wu et al. (2017) reported microcrystalline cellulose as a carbon precursor to prepare a facile and low-cost one-step hydrothermal treatment of CQDs [212]. The modification of these CQDs with ethylenediamine not only allowed nitrogen atoms on its surface but also introduced them into the carbon nuclear lattice to obtain better optical properties and higher quantum yield than CQDs. Fortunately, nitrogen doped CQDs (NCQDs) have a fluorescence emission intensity of 7.1 times stronger than the CQDs, and it can be explained by the reduction of epoxy, ether, and carboxyl groups on the surface of NCQDs via nucleophilic reactions with amidogen, thus forming a few non-radiative recombination centers in NCQDs. The amination and/or amidation also create radiative recombination centers, such as amine (–C–N–) and amide groups (R_2_-NCOR), which belong to electron-donating groups. These electron-donating groups and π-electrons that existed in the –C=N group have contributed to the improvement of the fluorescence intensity of a substance. All in all, these NCQDs successfully provided the highest quantum yield of 55%.

The synthesis of CDs using non-acid reagents can be further explored by attempting to use three different aniline compounds, i.e., 1,2,4,5-benzenetetramine tetrahydrochloride, 1,2,4-benzenetriamine dihydrochloride, and o-phenylenediamine, as carbon sources and ethanol as a solvent [217]. In an approach where more amino groups and a high nitrogen content are present in CDs-benzenetetramine tetrahydrochloride, the excitation and emission wavelengths all exhibited a redshift with an increase in the CD particle size. The quantum yield also greatly increased up to 30.2%. Additionally, the fluorescence intensity of the CDs was observed to gradually increase with the increasing amount of raw material from 0.005 g to 0.025 g. It is important to note that the number of amino groups and particle size of CDs have a great influence on the emission wavelength, while the amount of raw material influences the color and intensity of the fluorescence. In another study, the CDs were synthesized using three types of aldehydes, such as glutaraldehyde, nitrobenzaldehyde and benzaldehyde, via the solvothermal method [225]. The resulting CDs were then composited with chitosan, functionalized by a DNA probe and utilized as a detecting platform for rapid detection of microRNA-21. The function of CDs is further classified as a cross-linking agent and fluorescent donor, while chitosan plays an important role in the preparation of three-dimensional hydrogel frames.

**Table 9:** Summary of the synthesis of CDs from non-acid reagents.

Precursor		Technique	Properties			Year	Reference
Carbon Source	Passivation/Solvent		Particle Size	Fluorescence	Quantum Yield		
Graphite oxide	Nitric acid	Microwave-hydrothermal	TEM- 4 nm	λ_em_- 520 nm λ_ex_- 470 nm	2.72%	2011	[198]
Poly(ethylene glycol)	Sodium hydroxide and distilled water	Reflux method	TEM- 5 nm	λ_em_- bright blue λ_ex_- 350 nm λ_em_- cyan λ_ex_- 390 nm λ_em_- yellow λ_ex_- 470 nm λ_em_- red λ_ex_- 540 nm	-	2013	[199]
Polyethylene glycol 1500	Serine and glycerin	Microwave pyrolysis	-	-	-	2013	[200]
3-(3,4-dihydroxyphenyl)-L- alanine	-	Carbonization-oxidation	TEM- 3.64 nm	λ_em_- 500 nm λ_ex_- 400 nm	6.3%	2013	[201]
	Nitric acid		TEM- 4.31 nm	λ_em_- 475 nm λ_ex_- 360 nm	1%		
Ethanolamine	-	Pyrolysis	TEM- 2.7 nm	λ_em_- 450 nm λ_ex_- 365 nm	7%	2014	[202]
	Hydrogen peroxide		TEM- 8.3 nm		10.3%		
Polyimide	-	Hydrothermal	TEM- 4 nm	λ_em_- 490 nm λ_ex_- 365 nm	20.9%	2015	[203]
Polyethyleneglycol bis(3-aminopropyl)	6-Bromohexylboronic acid	Thermal carbonization	TEM- 5 nm	λ_em_- 440 nm λ_ex_- 362 nm	0.3%	2015	[204]
Ethanol	Hydrogen peroxide and deionized water	Hydrothermal	TEM- 4.8 nm	λ_em_- 456 nm λ_ex_- 400 nm	38.7%	2015	[205]
3-Bromophenol	Ethanol and deionized water	Carbonization	TEM- 5.2 nm	λ_em_- 440 nm λ_ex_- 367 nm	19.6%	2016	[206]
Azidoimidizole	Ethanol	-	AFM- 5–10 nm	λ_em_- 515 nm λ_ex_- 460 nm	-	2016	[207]
Activated carbon	Potassium permanganate, sulfuric acid, deionized water, hydrogen peroxide	Exhausted oxidation	TEM- 12 nm	λ_em_- 465 nm λ_ex_- 350 nm	3.94%	2016	[208]
	Potassium permanganate, sulfuric acid, deionized water, hydrogen peroxide, PAMAM-NH_2_		TEM- 65 nm		6.93%		
Glucose	Water and sodium hydroxide	Ultrasonic	-		7%	2016	[209]
N-Methylethanolammonium thioglycolate	Water and hydrogen peroxide		HRTEM- 3–8 nm		12.5%		
Chitosan	-	Carbonization	TEM- 1–6 nm	λ_em_- 390 nm λ_ex_- 310 nm	4.34%	2016	[210]
Chlorophyll	Water	Hydrothermal	DLS- 18 nm	λ_em_- 520 nm λ_ex_- 440 nm	-	2017	[211]
Microcrystalline cellulose	Ethylenediamine	Hydrothermal	TEM- 3.2 nm	λ_em_- 426–436 nm λ_ex_- 360 nm	55%	2017	[212]
o-Phenylenediamine	Ethanol	Hydrothermal	TEM- 1–2 nm	λ_em_- 400–600 nm λ_ex_- 350–500 nm	20%	2018	[213]
Carbon paper	Nitric acid	Hydrothermal	TEM- 4.8 nm	λ_em_- 450 nm λ_ex_- 350 nm	5.1%	2018	[214]
Papain and PEG6000	Ultrapure water	Hydrothermal	TEM- 2–3 nm	λ_em_- 420 nm λ_ex_- 320 nm	9.45%	2018	[215]
Dextrose solution	Hydrochloric acid	Mechano-chemical	TEM- 10 nm	λ_em_- 456 nm λ_ex_- 390 nm	40%	2018	[216]
1,2,4,5- Benzenetetramine tetrahydrochloride	Ethanol	Solvothermal	TEM- 9.39 nm	λ_em_- 605 nm λ_ex_- 540 nm	30.2%	2019	[217]
1,2,4-Benzenetriamine dihydrochloride			TEM- 8.60 nm	λ_em_- 598 nm λ_ex_- 510 nm	13.4%		
o-Phenylenediamine			TEM- 6.50 nm	λ_em_- 538 nm λ_ex_- 420 nm	16.7%		
Copper (II) chloride dihydrate	Ethanediamine	Hydrothermal	TEM- 1.8 nm	λ_em_- 380 nm λ_ex_- 320 nm	7.8%	2019	[218]
Glucose and taurine	Distilled water	Hydrothermal	TEM- 3 nm	λ_em_- 410 nm λ_ex_- 340 nm	11%	2019	[219]
Polyethylene glycol	-	Pyrolysis	DLS- 10 nm	λ_em_- ~380 nm λ_ex_- 340 nm	16%	2020	[220]
m-Phenylenediamine	Deionized water	Hydrothermal	TEM- 5.1 nm	λ_em_- 420 nm λ_ex_- 340 nm	12%	2020	[221]
Glucosamine	Ethylenediamine and water	Microwave digestion	TEM- 4.45 nm	λ_em_- 466 nm λ_ex_- 384 nm	25.38%	2020	[222]
Lactose	Hydrochloric acid	Hydrothermal	TEM- 7 to 8 nm	-	-	2021	[223]
Selenourea and o-phenylenediamine	Hydrochloric acid	Hydrothermal	TEM- 3 nm	λ_em_- 625 nm λ_ex_- 564 nm	23.6%	2021	[224]
Glutaraldehyde	Ethanol	Solvothermal	TEM- 1 nm	λ_em_- 453 nm λ_ex_- 360 nm	-	2021	[225]
Nitrobenzaldehyde			TEM- 5 nm	λ_em_- 421 nm λ_ex_- 360 nm			
Benzaldehyde			-	λ_em_- 430 nm λ_ex_- 360 nm			
Diphenyl ether	p-Phenylenediamine		TEM- 2.8 nm	λ_em_- ultraviolet λ_ex_- 285 nm	8%	2021	[226]
	p-Phenylenediamine, dopamine and tris(hydroxymethylaminomethane)		TEM- 10–18 nm	λ_em_- red λ_ex_- 285 nm	15.5%		
o-Phenylenediamine	Ethanol	Two separate solutions mixed in one-pot hydrothermal	TEM- 5 nm	λ_em_- ~570 nm λ_ex_- 430 nm	-	2022	[227]
Ammonium sulfate	Deionized water						
Glucose	Deionized water	Hydrothermal	TEM- 8.9 nm	λ_em_- 450 nm λ_ex_- 350 nm	-	2022	[228]
	Boric acid		TEM- 6.2 nm	λ_em_- 400 nm λ_ex_- 320 nm			
	Sodium persulfate		TEM- 6.9 nm	λ_em_- 400 nm λ_ex_- 320 nm			
	Urea		TEM- 5.6 nm	λ_em_- 450 nm λ_ex_- 390 nm			
p-Phenylenediamine and thylenediamine	Anhydrous ethanol	Hydrothermal	TEM- 2.76 nm	-	-	2022	[229]
m-Phenylenediamine	Ethanol	Solvothermal	TEM- 6.9 nm	λ_em_- 440 nm λ_ex_- 380 nm	11%	2022	[230]
o-Phenylenediamine			TEM- 7.8 nm	λ_em_- 550 nm λ_ex_- 380 nm	17%		

## 4. Application of CD-Based Optical Sensor for Environmental Monitoring

**Figure 6:**
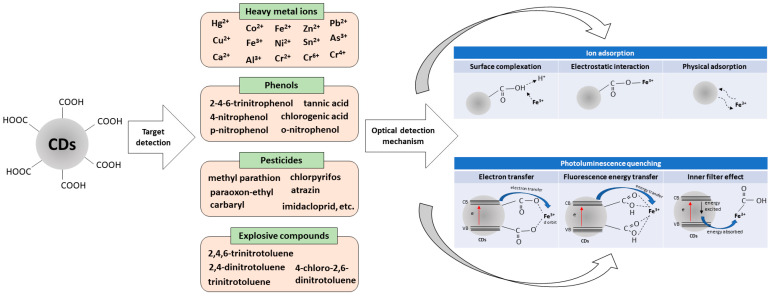
Schematic diagram of target detection and possible detection mechanism.

The incorporation of CDs in optical sensors for environmental monitoring, including the detection of heavy metal ions, phenol, pesticides, and nitroaromatic explosives, are reviewed below (Figure 6). This review aims to survey state-of-the-art CD-based optical sensors with their sensing performance, namely range of detection, limit of detection, and linear correlation coefficient.

### 4.1. Heavy Metal Ions

Environmental pollutions caused by toxic heavy metal ions (HMI) have resulted in the development of different optical sensors using nanoscale materials [231,232,233,234,235,236,237]. Of late, CD-based optical sensors have garnered tremendous research interest, which is summarized chronologically in Table 10. Herein, an optical fibre sensor for Hg^2+^ and Cu^2+^ detection was developed using CDs functionalized with poly(ethylene glycol) and N-acetyl-l-cysteine [238]. This sensor has quite interesting analytical potential, as it allows for higher linear coefficients when detecting Hg^2+^ in aqueous solutions. Furthermore, the concentration range of Hg^2+^ was successfully lowered to femtomolar detection using a fluorescent sensor [199]. The outstanding nature of the sensing performance can be probably attributed to the Hg^2+^ ions, which have stronger affinity toward carboxylic groups on the surface of CDs and stronger quenching effect on the PL of the prepared CDs.

On the other hand, Co^2+^ and Cu^2+^ ions have been detected using CD-based composites. In the presence of CTAB to PEG-passivated CDs, a long fluorescence lifetime with higher emission efficiency was noticed due to the rapid production of •OH radicals from the Co(II)-H_2_O_2_-OH^−^ system, which is responsible for the highly selective response of the present chemiluminescence system towards Co^2+^ ions [200]. On the other hand, the electrochemiluminescence of the oxidized CDs/K_2_S_2_O_8_ system resulted in higher quenching to 20 nM of Cu^2+^ compared to 1 µM of Pb^2+^, Ni^2+^, Mn^2+^, Fe^2+^, and Co^2+^ [201]. Another HMI of interest is Fe^3+^, which have been detected three times in a row in the year of 2014 using diverse optical sensors [116,117,119]. Among them, the phenolic hydroxyl of N-doped CDs exhibited higher sensitivity toward Fe^3+^ quenching, with the linearity range of 0.01−500 μM and detection limit of 2.5 nM [116]. The reason is that the hydroxyl group has a good binding affinity for Fe^3+^, leading to the splitting of d orbital of Fe^3+^ and consequently causing significant fluorescent quenching. Similarly, nitrogen-doped CQDs were used as an effective fluorescent sensing platform for label-free sensitive detection of Hg^2+^ ions in an ultrapure water solution with a detection limit of 0.23 μM [118]. Due to the excellent sensitivity and selectivity of the Hg^2+^ sensor, this sensor was further applied to the determination of Hg^2+^ in tap water and real lake water samples. The results showed that the PL intensity (excited at 360 nm) decreased gradually with the increasing concentration of Hg^2+^ in both tap water and lake water from 5 to 50 μM.

In other work, the sensing potential of fluorescent CDs synthesized through the pyrolysis method was evaluated for Cu (II), Cr (II), Co (II), Ni (II), Al (III), Ca (II), Pb (II), Zn (II), Sn (II) and Hg (II) ions [79]. Among all the metal ions tested, Cu (II) and Pb (II) have the highest sensitivity, while Zn (II), Hg (II) and Ca (II) have the lowest sensitivity. The reason for this relatively low sensitivity might be due to the diamagnetic properties of Zn (II), Hg (II) and Ca (II) ions, which eliminate the paramagnetic quenching mechanism with the CDs.

With the accelerating demand of mercury in the skin lightening industry, a high number of optical techniques for Hg^2+^ have been reported [15,23,54,65,89,122,124,125,126,127,128,133,142,143,152,153,160,178,207]. Notably, fluorescence techniques are the most employed. Altogether, there are several phenomena about the quenching effect of CDs to Hg^2+^, which are as follows: (i) electron transfer from excited nitrogen and sulfur atoms in CDs to Hg^2+^ (nonradiative recombination); (ii) strong binding between Hg^2+^ and S atoms or carboxylate or hydroxyl groups from CDs; and (iii) Hg^2+^ has a larger ionic radius and polarization; therefore, deformation happened more easily when it interacted with the nitrogen and sulfur atoms. Fortunately, the Hg^2+^ can be detected as low as 2.6 nM using CD-labeled oligodeoxyribonucleotide (ODN) and quenched by graphene oxide [122]. In this study, CD-ODN acted as the energy donor and molecular recognition probe and GO served as the fluorescence resonance energy transfer acceptor.

Due to the concerns about improving and protecting living organisms, more optical detection methods that utilize CDs as a sensing element have been developed to detect Fe^2+/3+^ [13,22,38,40,72,78,87,124,135,142,151,159,166,169,219,226], Cu^2+^ [53,66,97,141,227,239], Pb^2+^ [65,87,181,240], Cr^6+/4+^ [23,85,193,241], and Co^2+^ [51]. For instance, Shamsipur et al. (2018) prepared green-emitting CDs instead of common blue-emitting CDs for selective and sensitive detection of Fe^3+^ [159]. This green CDs approach could detect Fe^3+^ in the range of 0.05–10.0 μM, with a detection limit of 0.0137 μM. The detection limit was found to be significantly lower than the maximum level of Fe^3+^ (0.54 μM) allowed in drinking water by the U.S. Environmental Protection Agency.

**Figure 7:**
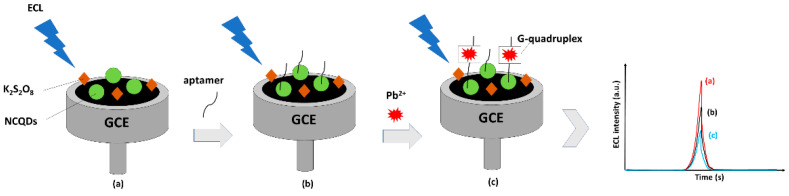
Surface functionalization of (**a**) GCE/NCQDs, (**b**) GCE/NCQDs/aptamer, and (**c**) GCE/NCQDs/aptamer/Pb^2+^.

Li et al. (2021) invested in interesting research to detect low concentrations of Pb^2+^ ions [181]. An electrochemiluminescence (ECL) aptasensor was prepared by modifying a glassy carbon electrode (GCE)/NCQDs with amino aptamers by NHS/EDC linkage for selective and sensitive detection of Pb^2+^. As shown in Figure 7, the binding of the hairpin aptamer with Pb^2+^ forms the G-quadruplex, and thus exposes the amino group to the NCQDs, causing significant changes in ECL intensity. The results revealed that the GCE/NCQDs/aptamer sensor can detect Pb^2+^ quickly and accurately, providing the lowest detection limit of 18.9 pM. Importantly, the proposed aptasensor did not suffer from interference and it had excellent stability.

The fabrication of CDs with industrial/agricultural waste has proven to be useful as a detection probe for metal ions. A study conducted by Yan et al. (2020) demonstrated that the synthesized N-CDs from crown daisy leaves had abundant surface functional groups that could selectively and sensitively detect Cu^2+^ at a very low detection limit of 1.0 nM by the fluorescent quenching effect [53]. This result is due to the selective complexation interaction between Cu^2+^ and carboxyl and amino groups of the N-CDs.

On the other hand, improving the detection performance of hexavalent chromium (Cr^6+^) even at the nanomolar level has become an enduring concern of researchers. Rajendran and Rajendiran (2018) reported that the CQDs can be effectively used as fluorescent probes for the detection of Cr^6+^ at the nanomolar level [23]. This research reported strong fluorescence quenching with increasing Cr^6+^ concentration, resulting in an improved detection limit of 10 nm. Nevertheless, the detection limit of Co^2+^ was found to be higher at the micromolar level despite using CD-based optical sensors [51]. The proposed sensor was then applied in the analysis of real river water samples with a recovery value of 95.0–106.8% and a relative standard deviation below 5.3%, indicating that the synthesized CDs had great application prospects in Co^2+^ detection.

**Table 10:** Summary of the developed optical sensors for heavy metal ions detection.

Heavy Metal Ions	Material	Optical Sensor	Range of Detection	Limit of Detection	Linear Correlation Coefficient	Year	Reference
Hg^2+^	CDs@PEG and N-acetyl-l-cysteine	Optical fibre	0–2.69 μM	-	0.977	2010	[238]
Cu^2+^					0.975		
Hg^2+^	CDs	Fluorescent	0–5 fM	1 fM	-	2013	[199]
Co^2+^	CTAB@CDs	Chemiluminescent	1.0–1000 nM	0.67 nM	0.992	2013	[200]
Cu^2+^	o-CDs/K_2_S_2_O_8_	Electrochemiluminescent	0–4 nM	-	-	2013	[201]
Fe^3+^	N-CDs	Chemiluminescent	1.0 × 10^−7^–1.0 × 10^−6^ M	66.7 nM	0.993	2014	[117]
Fe^3+^	N-doped CDs	Fluorescent	0.01–500 μM	2.5 nM	-	2014	[116]
Fe^3+^	CDs	Electrochemiluminescent	5–80 μM	700 nM	0.993	2014	[119]
Hg^2+^	N-CQDs	Fluorescent	0–25 μM	0.23 μM	0.994	2014	[118]
Pb^2+^	CDs	Fluorescent	0–47.62 μM	7.49 μM	-	2014	[79]
Cu^2+^				7.78 μM			
Al^3+^				13.38 μM			
Ni^2+^				13.90 μM			
Co^2+^				18.07 μM			
Cr^2+^				23.69 μM			
Sn^2+^				31.51 μM			
Ca^2+^				34.79 μM			
Hg^2+^				38.02 μM			
Zn^2+^				69.64 μM			
Fe^3+^	CDs	Fluorescent	0.10–10 μM	31.5 nM	0.9977	2015	[124]
Hg^2+^			0.01–2.0 μM	15.3 nM	0.9977		
Hg^2+^	CDs	Fluorescent	0.01–10 μM	3.3 nM	0.997	2015	[128]
Hg^2+^	ODN-CDs	Fluorescent	5–200 nM	2.6 nM	0.974	2015	[122]
Hg^2+^	N-S-CDs	Fluorescent	0–40 μM	2.0 μM	0.994	2015	[125]
Hg^2+^	N-CDs	Fluorescent	0–8 μM	0.087 μM	0.9962	2015	[127]
Hg^2+^	N,S-co-doped CDs	Fluorescent	0–20 μM	0.18 μM	0.9975	2015	[126]
Hg^2+^	N-rich CDs	Fluorescent	0–20 μM	0.63 μM	0.989	2016	[207]
Fe^3+^	N-doped CDs	Fluorescent	0–1000 μM	100 μM	-	2016	[135]
Fe^3+^	N-CDs	Fluorescent	0–1000 μM	0.96 μM	-	2016	[13]
Hg^2+^	LR-CDs	Fluorescent	0.1–1.5 μM	18.7 nM	0.9919	2016	[64]
			2.0–60.0 μM		0.994		
Hg^2+^	CDs	Fluorescent	0–80 μM	0.201 μM	0.9982	2016	[133]
Pb^2+^	CDs	Fluorescent	0.01–1.0 μM	0.59 nM	0.998	2017	[65]
Cu^2+^	Nitrogen-doped CDs	Fluorescent	0.5–4 μM	0.38 μM	0.998	2017	[141]
Hg^2+^	CDs	Fluorescent	0–0.5 mM	0.78 μM	0.9944	2017	[142]
Fe^3+^			0–0.15 mM	1.17 μM	0.9977		
Hg^2+^	Nitrogen-doped CQDs	Fluorescent	0–18 μM	83.5 nM	0.9979	2017	[143]
Hg^2+^	CDs	Fluorescent	0–40 μM	9 nM	0.9896	2017	[15]
Fe^3+^	N-CQDs	Fluorescent	0–300 μM	0.16 μM	0.9811	2018	[22]
Cr^6+^	CDs	Fluorescent	0–100 μM	0.73 μM	0.9903	2018	[85]
Cu^2+^	CQDs	Fluorescent	1–8 μM	6.33 nM	0.998	2018	[97]
Cu^2+^	CDs	Fluorescent	0.01–500 μM	4.3 nM	0.9907	2018	[66]
Cu^2+^	CQDs	Fluorescent	0–100 μM	31.5 μM	0.9897	2018	[239]
Fe^3+^	CdSe@SiO_2_-CDs	Fluorescent	9–120 μM	0.26 μM	0.995	2018	[151]
Fe^3+^	CDs	Fluorescent	0.05–10.0 μM	13.7 nM	0.992	2018	[159]
Hg^2+^	N-CDs	Fluorescent	0.001–5 μM	0.65 μM	0.985	2018	[160]
Hg^2+^	CDs	Fluorescent	0–100 μM	2.47 μM	0.9892	2018	[152]
Hg^2+^	N-S-CDs	Fluorescent	0.01–50 μM	0.008 μM	0.9622	2018	[153]
Hg^2+^	CQDs	Fluorescent	5–70 nM	8 nM	0.9970	2018	[23]
Cr^6+^				10 nM	0.9956		
Fe^3+^	CDs	Fluorescent	1–700 μM	<1 μM	0.993	2019	[219]
Fe^2+^	CDs	Optical microfiber	0–5.372 μM	0.179 μM	-	2019	[166]
Co^2+^	CDs	Fluorescent	1–2 μM	0.39 μM	0.9912	2019	[51]
Pb^2+^	VV-CDs	Fluorescent	1–100 μM	12 nM	0.99853	2020	[87]
Fe^3+^				16 nM	0.99933		
Pb^2+^	GCE/NCQDs/aptamers	Electrochemiluminescence	50–387.9 nM	0.0189 nM	0.998	2020	[181]
As^3+^	CDs-MnO_2_	Fluorescent	0–200 nM	16.8 nM	0.992	2020	[177]
Cu^2+^	CDs	Fluorescent	0–120 nM	1.0 nM	0.997	2020	[53]
Fe^3+^	Phe-CDs	Fluorescent	5–500 μM	0.720 μM	0.9959	2020	[169]
Hg^2+^	N-CDs	Fluorescent	0.15–90 μM	0.20 μM	0.993	2020	[178]
Hg^2+^	CDs	Fluorescent	0.01–5 μM	6.25 nM	0.991	2020	[54]
Cr^4+^	S, N-CDs	Fluorescent	0.03–50 μM	21.14 nM	0.996	2021	[193]
Fe^3+^	N-CDs	Fluorescent	0.3–3.3 μM	0.135 μM	0.9918	2021	[72]
Fe^3+^	CDs@PDA	Fluorescent	2–27 μM	3.75 μM 5.82 μM	0.994 0.991	2021	[226]
Pb^2+^	N-CDs/R-CDs@ZIF-8	Fluorescent	0.05–50 μM	4.78 nM	0.9952	2021	[240]
Cr^6+^	BNCDs	Fluorescent	0–100 μM	0.41 μM	0.999	2021	[241]
Fe^3+^	KBNCDs	Fluorescent	0–25 μM	1.2 μM	0.997	2022	[78]
Mn^2+^				1.4 μM	0.998		
Hg^2+^	CDs	Fluorescent	0–46 μM	2 μM	0.997	2022	[89]
Fe^3+^	CDs	Fluorescent	20–100 μM	0.07 μM	0.9977	2022	[38]
Fe^3+^	M-CDs	Fluorescent	5–30 μM	0.47 μM	0.998	2022	[40]
Cu^2+^	NS-CDs	Colorimetric	1–100 μM	200 nM	0.99481	2022	[227]

Where CDs@PEG: carbon dots functionalized poly(ethylene glycol), CTAB: cationic cetyltri-methylammonium bromide, o-CDs/K_2_S_2_O_8_: oxidized carbon dots/potassium persulfate, N-CDs: nitrogen doped carbon dots, N-CQDs: nitrogen doped carbon quantum dots, ODN-CDs: carbon dots-labeled oligodeoxyribonucleotide, N,S-CDs: nitrogen and sulphur co-doped carbon dots, LR: lotus root, CdSe@SiO_2_-CDs: silica coated cadmium selenide carbon dots, VV-CDs: *Volvariella volvacea*-carbon dots, GCE: glassy carbon electrode, MnO_2_: manganese dioxide, Phe-CDs: phenylalanine-carbon dots, CDs@PDA: p-phenylenediamin-derived carbon dots, N-CDs/R-CDs@ZIF-8: nitrogen-doped carbon dots/red-carbon dots@zeolite imidazole, BNCDs: boron-nitrogen co-doped carbon dots, KBNCDs: Kentucky bluegrass nitrogen-doped carbon dots, M-CDs: *Morus nigra* carbon dots, Hg^2+^: mercury ions, Cu^2+^: copper ions, Co^2+^: cobalt ions, Fe^3+^/^2+^: ferric ions, Pb^2+^: lead ions, Al^3+^: aluminum ions, Ni^2+^: nickel ions, Cr^2+^/^4+^/^6+^: chromium ions, Sn^2+^: tin ions, Ca^2+^: calcium ions, Zn^2+^: zinc ions, As^3+^: arsenic ions.

### 4.2. Phenols

The existence of different forms of phenolic in petrochemical engineering, printing, food processing, and dyeing has been continuously reported as the most harmful pollutant due to its high toxicity and low biodegradability [242]. Table 11 summarizes the chronological order of the detection of phenolic compounds using optical sensors. Among the reported studies, 2-4-6-trinitrophenol, also called picric acid, is the most common pollutant monitored by means of fluorescent sensor [72,112,114,134,136,150,170,221]. It is well known that 2-4-6-trinitrophenol is a representative electron-deficient nitroaromatic, due to the electron-withdrawing nature of the three nitro groups. From the perspective of the sensor performance, Niu et al. (2013) explored the feasibility of using amine-capped CDs for the detection of 2-4-6-trinitrophenol in aqueous and ethanol solutions [114]. For the detection of 2-4-6-trinitrophenol in ethanol, the amine-capped CDs demonstrated obvious fluorescent quenching and superior sensitivity to 2-4-6-trinitrophenol in aqueous solution. Since water is much more polar than ethanol, the detection is, therefore, driven by an electrostatic interaction between 2-4-6-trinitrophenol and CDs.

Generally, there are three possible mechanisms causing fluorescence quenching between the phenolic compounds and proposed CDs, which are as follows: (i) electron transfer; (ii) fluorescence energy transfer; and (iii) inner filter effect. Amid all the mechanisms, fluorescence energy transfer (FET) is a dominant quenching pathway due to the electron-rich nature of the CDs and the electron-deficient aromatic group of the phenol [115,134,243]. In contrast to FET, the electron transfer process became possible for phenol fluorescence quenching [112,150,204]. Notably, electron transfer in the excited state of CDs can occur from the lowest unoccupied molecular orbital (LUMO) to the LUMO of phenols, if possible. In addition, there are certain rules that are required to fulfil FET quenching. The rules are as follows: (i) the absorption spectrum of the quencher (phenols) should overlap with the emission spectrum fluorophore and (ii) there is a change in the fluorescence lifetime of CDs before and after the addition of the quencher. Furthermore, the inner filter effect (IFE) is also considered important and has been applied as a developing novel fluorescent assay in recent studies [34,136,170,218,219,226]. This approach offers more simplicity and expediency because IFE-based sensors use fluorophore and the receptor directly rather than chemically interacting with each other.

In another study, Xue et al. (2018) found that phenol can form hydrogen bonds with the carboxyl group on the surface of the fluorescence CQDs, which will facilitate the fluorescence quenching of the CQDs in the excited state, leading to the high sensitivity and selectivity of CQDs for the detection of phenol [95]. In addition, the fluorescence quenching effect can be explained by either a dynamic or static process. Interestingly, a static quenching effect between N-CDs and tannic acid found in the works of [167] can be proved by several measurements, which are as follows: (i) a decrease in the fluorescence lifetime; (ii) a rate that is constant more than 1.0 × 10^10^ M^−1^s^−1^; and (iii) a blue-shift of the absorption peak of CDs. Wang et al. (2019) compared the selectivity of the CDs@MIP and CDs@MIP to 4-nitrophenol, which resulted in good selectivity of CDs@MIP on 4-nitrophenol, due to the hydrogen bonding interactions, leading to the charge transfer from CDs to 4-nitophenol [165]. There is still much room for further development of CD-based optical sensors for the detection of phenolic compounds, despite achieving a high quantum yield and good sensing performance. For instance, Saravanan et al. (2020) successfully synthesized N@CDs from m-phenylenediamine as a single source of carbon and nitrogen to detect 2-4-6-trinitrophenol [221]. The fluorescent N@CD probe showed high selectivity toward 2-4-6-trinitrophenol relative to other nitro explosives, such as 4-nitrophenol, 4-amino-3-nitrophenol, 2,5-dinitrophenol, phenol, 2-chlorophenol, 3-nitrophenol, and 4-amino-triphenol.

In addition, ultra-weak chemiluminescent (CL) systems have become one of the focuses of the increasing attention on the determination of tannic acid [173]. Interestingly, tannic acid could dramatically suppress the CL intensity of the FNCDs-H_2_O_2_-K_3_Fe(CN)_6_ system with an excellent linear response (R^2^ = 0.9971) and was further applied for the determination of tannic acid in a red wine sample. The recovery of 98.9–102.1% was obtained, demonstrating its reliability and application potential in real sample analysis. Recently, the aggregated N,S-CDs as a dual-excitation ratiometric fluorescent probe have proven to be an effective approach for the quantitative determination of chlorogenic acid [184]. Apart from dual excitation, Liu et al. (2021) observed the dual-color fluorescence emission of ultraviolet and red light when detecting 4-nitrophenol using CDs@PDA [226]. The detection limits obtained for the ultraviolet and red-light emission were 3.44 µM and 7.29 µM, respectively. In addition, 2-4-6-trinitrophenol has been detected using the fluorescence method with a detection limit of 0.11 μM in the concentration range of 0.3–3.3 μM [72]. The possible mechanism for fluorescence quenching of N-CDs by 2-4-6-trinitrophenol is explained by the electrostatic interaction or hydrogen bonding that occurs between the nitrogen groups of CDs and phenolic group of 2-4-6-trinitrophenol. To be exact, the electrostatic interactions occur when a large number of nitro groups with electron-withdrawing characteristics on benzene interact with nitrogen-containing functional groups of CDs. Hence, it can be concluded that nitro groups play a vital role in fluorescent quenching.

### 4.3. Pesticides

Pesticides are chemical compounds that are used to kill pests. Generally, there are six classes of pesticides according to their target species, i.e., insecticides, herbicides, rodenticides, bactericides, fungicides, and larvicides. The largest and most widely used in crop protection are insecticides [244]; however, they are classified as an extremely toxic class of chemical compounds by the World Health Organization (WHO).

Organophosphorus (OP) compounds, such as methyl parathion [121,138], paraoxon-ethyl [137], dichlorvos [245], malathion [245], ethion [245], paraoxon [154,155,211], chlorpyrifos [63,96,246], methyl-paraoxon [180], diazinon [50,62], and quinalphos [247], [63], are widely reported insecticides in the development of CD-based optical sensors. Oddly, most OP pesticides are always selected as inhibitors for sensors based on enzyme activity. According to Hou et al. (2016), dichlorvos can inhibit the activity of acetyl cholinesterase (AChE), which can turn the fluorescence of CDs off again [245], which is similar to the detection of paraoxon [155]. However, in a study of Lin et al. (2017), only OP of chlorpyrifos effectively inhibited H_2_O_2_ production by destroying the acetylcholinesterase activity, thereby increasing the fluorescence of C-dots after being initially turned off by Fe^3+^ [96]. Moreover, the proposed sensor exhibits a low selectivity against OP. Therefore, a new facile fluorescence probing based on novel N-doped carbon dots and methyl parathion hydrolase (MPH) was developed to detect methyl parathion selectively [138]. The use of MPH in this study helps to degrade OP compounds and has many advantages over other enzymes, such as less background noise and high turnover number, which is favourable for sensitive detection.

Another class of insecticide is carbamates. Li and co-workers have been devoted to detecting carbaryl in the presence of acetylcholinesterase (ACh) and choline oxidase (ChOx) by using N-CQDs, S-CQDs, and co-doped N, S-CQD-based photoluminescence sensors [209]. The results demonstrated that high sensitivity originates from the N and S dopants, which offer stronger capacities to adsorb hydrogen peroxide (H_2_O_2_) and generate local states to trap hot electrons, promoting the electron transfer to H_2_O_2_, and thus resulting in the strong quenching of the CQD fluorescence.

Apart from the insecticide organophosphate, insecticides neonicotinoids and organochlorine have become a target pesticide. Mandal et al. (2019) have reported a simple fluorescence-based method using CDs for the detection of commonly used insecticides, such as imidacloprid and lindane [246]. The fluorescence emission of the CDs was found to be enhanced in the presence of the increasing concentration of imidacloprid; however, the emission was quenched in the presence of lindane. The interactions between CDs and insecticides were mainly driven by the high reactive -NO_2_ and -NH groups in imidacloprid that directly bind to the surface functionality amino groups of CDs and by the H-bond or week ionic interaction or covalent binding between the free surface groups of the CDs and lindane, followed by the substitution reaction. In addition, the researchers also expanded the potential of CDs in sensing unclassified pesticides, namely tetradifon [246]. The leaving groups (-Cl) of tetradifron were found to possessstrong covalent binding with the surface amine group of the CDs with a high affinity. Nonetheless, the results showed that tetradifron has a higher detection limit than imidacloprid, but offers a lower detection limit than lindane. This phenomenon can be attributed to the binding affinity of all the different target pesticides. Their binding affinity can be arranged in the following decreasing order: imidacloprid > tetradifron > lindane.

Until 2021, the fluorescence response of CDs towards herbicides [50,60,213,220,246] and fungicides [73,182,183] has also been explored. It is reported that with increasing concentrations of herbicides, i.e., atrazine [213], pretilachlor [60], glyphosate [50], and fungicide thiophanate methyl [183], this has resulted in an increase in PL emission intensity (turn-on). This fluorescence ‘turn-on’ model was dynamic quenching, whereas, when the concentrations of trifluralin [220], pyrimethanil [182], and isoprothiolane [73] were increased, the fluorescence emission of CDs was found to decrease, leading to static quenching. Supchocksoonthorn et al. (2021) suggested that the fluorescent quenching is closely related to the inner filter effect, due to the π-π interaction between the large aromatic core of the CDs and anilinopyrimidine unit of pyrimethanil and between their polar functional groups [182].

**Figure 8:**
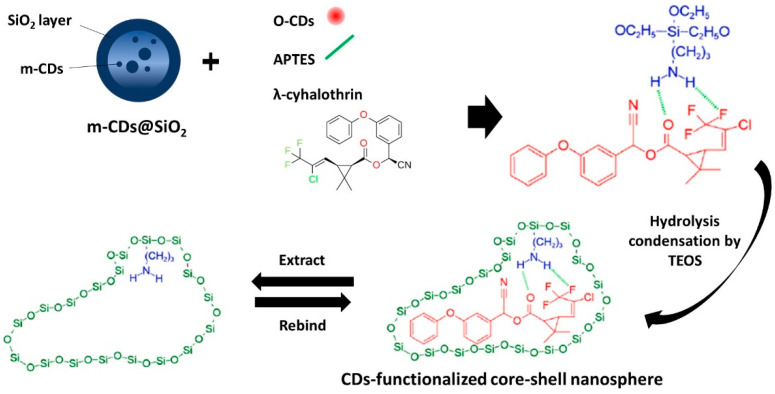
Schematic diagram of binding reactions of the prepared CD-functionalized core-shell nanospheres for detection of λ-cyhalothrin.

Furthermore, as an improvement to the method with a single fluorescence signal, Zhu et al. (2022) developed a new type of CD-functionalized core-shell nanospheres as a ratiometric fluorescence to detect λ-cyhalothrin (LC), a typical pyrethroid insecticide. Figure 8 shows the main binding reactions in the preparation of the CD-functionalized core-shell nanospheres. Here, silica nanoparticles play a role in preventing the contact between m-CDs and the dispersion solvent so the m-CDs could act as a reliable reference background; o-CDs act as a fluorescence signal; 3-aminopropyrtriethoxy silane (APTES) as a functional monomer to connect with LC through hydrogen bonding; ionic liquids (IL) as a cationic surfactant, which are attached to the system by electrostatic interaction to enhance the fluorescence of the core-shell nanospheres; and tetraethyl orthosilicate (TEOS) is used to form a layer of imprinted silicon shell on the surface of m-CDs@SiO_2_. Inspired by the role of materials, these newly formed core-shell nanospheres managed to achieve lower detection limits, excellent reusability, and better precision than those of other methods reported in this work [230]. All the proposed CD-based optical sensors for the detection of pesticides are tabulated orderly in Table 12.

### 4.4. Explosive Compounds

Military-grade explosives such as trinitroluene (TNT), dinitroaniline (DNT), are still a major worldwide concern in terms of terror threat and environmental impact. The only method that detects explosive compounds using CDs is a fluorescent sensor. For instance, Zhang et al. (2015) used N-rich CDs for 2,4,6-trinitrotoluene determination [120]. Apparently, TNT is a typical electron deficient nitro compound, which can selectively interact with amine group on CDs, leading to charge transfer from fluorescent CDs to the aromatic rings of TNT and consequently quenching the fluorescence emission of CDs strongly.

Dai and co-workers found that fluorescence sensors are well suited for detecting nitroaromatic compounds, such as 2,4-dinitrotoluene (DNT), as they can quench the emission of the excited species [132]. In this work, static quenching occurred with an increasing concentration of DNT, as CDs formed stable charge-transfer complexes with DNT molecules. Considering that the selectivity of those sensors were relatively low because some metal ions or other structure analogy can quench those fluorescent nanoparticles, Xu et al. introduced molecularly imprinted polymers (MIPs) to improve the selectivity [131]. However, this causes poor sensitivity because of the highly cross-linked nature of MIPs. Therefore, a novel strategy using periodic mesoporous silica particles as the imprinting matrix and using amino-CDs directly as a “functional monomer” was proposed to improve the sensitivity of M-MIPs@CDs.

In addition, Campos et al. (2016) first demonstrated the fluorometric sensing of 4-chloro-2,6-dinitroaniline in an aqueous solution using carbon quantum dots coated with PAMAM-NH_2_ [208]. The presence of amine groups in the PAMAM-NH_2_ dendrimer results in the enhanced fluorescence intensity and quantum yield from 3.94% to 6.93%. Moreover, it is interesting that with an increase in pH starting from 2.25, the fluorescence intensity increases drastically and then become constant from pH 6 to 11. The reason for the constant fluorescence intensity may be due to the buffering effect of the surface groups (–COOH and –NH_2_ mainly).

Next, a selective and sensitive detection of TNT explosive residues based on CD-modified optical sensors has been developed and rapidly elevating until 2022 [99,139,196,214,248]. The research concluded that the possible mechanism for TNT detection corresponds to the Jackson–Meisenheimer (JM) complex. Particularly, functionalizing CDs with amino groups can selectively form a JM complex with typical electron-deficient groups in TNT via charge transfer during a nucleophilic aromatic substitution reaction, which consequently causes fluorescence quenching. In addition, the selectivity of the sensor against TNT was investigated by Devi’s group [214]. The proposed sensor showed high selectivity for TNT over other weaker Lewis acids, such as dinitrotoulene (DNT), 2,4-dinitrotoluic acid (TA), nitrobenzene (NB), and p-nitrophenol (NP). It can be concluded that the JM complex formed by TNT is more stabilized due to more resonance structures; more nitro groups on the TNT aromatic ring than that formed by DNT, TA, NB, and NP. All the proposed CD-based optical sensors for the detection of explosive compounds are tabulated orderly in Table 13.

## 5. Concluding Remarks

An enormous research effort has been devoted to the development of CDs using green and chemical precursors. The reason behind the interest in this fabrication is its fluorescent properties that make it continuously competitive to produce bright and high-quality CDs. This paper has presented the recent progress in the field of CDs, focusing on the green and chemical precursors and fluorescent properties. To the best of our knowledge, this is the first review paper that highlights each of the precursors used in the CDs with the values of the fluorescence quantum yield.

**Figure 9:**
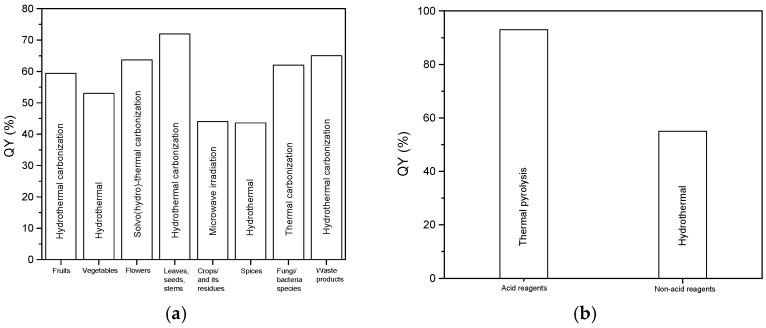
Quantum yields of the CDs based on (**a**) green precursors and (**b**) chemical precursors.

Among the available green precursors, CDs produced by plants are the most popular source because it is a more stable and straightforward process to scale up. For instance, the CDs synthesized from leaves, i.e., *Calotropis procera* leaves, without any addition of surface passivation have the highest quantum yield of 71.95% under the hydrothermal carbonization process, as shown in Figure 9a. It can be observed that the leaves have shown great potential as carbon sources due to their carbon-, hydrogen-, oxygen-, nitrogen-rich composition, which can eliminate the need of surface-chemical passivating agents. Other factors that contributed to the excellent photoluminescence properties include (i) heteroatom doping, such as sulfur, nitrogen, phosphorus, and boron; (ii) heating temperature and time; (iii) type and ratio of solvent; and (iv) pH of media. Even so, the quantum yields obtained from the green precursors were still low compared to the chemical precursors, as depicted in Figure 9b. It was shown that the thermal pyrolysis treatment of sodium citrate dihydrate and urea led to green fluorescence CDs with a quantum yield as high as 93%. As expected, CDs synthesized from acid reagents possess higher quantum yields due to the existence of carboxyl groups. The greater the number of carboxyl groups in the carbon source, the more amino groups of urea that will conjugate onto the surface of CDs.

Although the highest quantum yield results have been achieved using chemical precursors (acid reagents), the high toxicity of this precursor may hinder their potential for diverse applications. Therefore, future development should firstly focus on synthesizing CDs from green precursors, particularly plant parts and waste products. In addition, it is highly necessary to fabricate green precursors consisting of hydroxyl, carboxyl and amino groups to avoid the use of toxic chemical passivating agents. Furthermore, this will allow for the development of high-quality and non-toxicity CDs, finally paving the way towards their practical application in various fields.

## Figures and Tables

**Table 1:** Summary of the synthesis CDs from fruits.

Precursor		Technique	Properties			Year	Reference
Carbon Source	Passivation/Solvent		Particle Size	Fluorescence	Quantum Yield		
Orange juice	-	Hydrothermal	TEM- 2.5 nm	λ_em_- 441 nm λ_ex_- 360 nm	26%	2012	[9]
Watermelon peels	-	Carbonization	TEM- 2.0 nm	λ_em_- 490–580 nm	7.1%	2012	[10]
Sugar cane bagasse	Sodium hydroxide solution	Hydrothermal carbonization	HRTEM- 1.8 nm	λ_em_- 475 nm λ_ex_- 370 nm	12.3%	2014	[11]
Lemon peels	Sulfuric acid	Hydrothermal carbonization	TEM- 1 to 3 nm	λ_em_- 441 nm λ_ex_- 360 nm	14%	2016	[12]
Prunus avium extract	Ammonia	Hydrothermal carbonization	HRTEM- 7 nm	λ_em_- 411 nm λ_ex_- 310 nm	13%	2016	[13]
Cornstalk	Distilled water	Hydrothermal	TEM- 5.2 nm	λ_em_- 500 nm λ_ex_- 420 nm	7.6%	2017	[14]
Corn bract	Anhydrous ethanol	Solvothermal	TEM- 1.8–3.4 nm	λ_em_- 470 nm and 678 nm λ_ex_- 406 nm	6.9%	2017	[15]
Dried lemon peel	Deionized water	Hydrothermal	TEM- 9.5 nm	λ_em_- 505 nm λ_ex_- 425 nm	11%	2017	[16]
Pulp-free lemon juice	Ethanol	Solvothermal	AFM- 1.5 nm TEM- 4.6 nm	λ_em_- 631 nm λ_ex_- 540 nm	28%	2017	[17]
Citrus lemon peels	-	Carbonization	TEM- 4.5 nm	λ_em_- 435 nm λ_ex_- 330 nm	16.8%	2017	[18]
Citrus sinensis peels			TEM- 6.5 nm	λ_em_- 455 nm λ_ex_- 365 nm	15.5%		
Lemon juice	-	Thermal decomposition	-	λ_em_- 400 nm λ_ex_- 320 nm	7%	2018	[19]
Lemon juice	Poly(ethylenimine)	Carbonization	TEM- 5.7 nm	λ_em_- 540 nm λ_ex_- 420 nm	-	2018	[20]
Citrus lemon juice	-	Hydrothermal	TEM- 5.8 nm	λ_em_- 450 nm λ_ex_- 360 nm	10.20%	2018	[21]
Watermelon juice	Ethanol	Hydrothermal	TEM- 3–7 nm	λ_em_- 439 nm λ_ex_- 355 nm	10.6%	2018	[22]
Jackfruit juice	Ethanol and distilled water	Hydrothermal	HRTEM- <2.5 nm	λ_em_- 485 nm λ_ex_- 395 nm	14.6%	2018	[23]
Lemon	Ethylenediamine	Hydrothermal	TEM- 20 nm	-	20%	2018	[24]
Grapefruit							
Durian juice	Water and ethanol	Carbonization	-	-	-	2018	[25]
Acerola fruit	Water	Hydrothermal	-	λ_em_- 504 nm λ_ex_- 360 nm	-	2019	[26]
Bitter orange juice	-	Hydrothermal	AFM- 2–4 nm DLS- 1–2 nm	λ_em_- 390 nm λ_ex_- 325 nm	19.9%	2019	[27]
Citrus lemon juice	Ethylenediamine	Hydrothermal	HRTEM- 3 nm	λ_em_- 452 nm λ_ex_- 360 nm	31%	2019	[28]
Lemon and onion juices	Ammonium hydroxide solution	Microwave assisted carbonization	TEM- 6.15 nm	λ_em_- 425 nm λ_ex_- 340 nm	23.6%	2019	[29]
Lemon juice	-	Hydrothermal	HRTEM- 3–15 nm	λ_em_- 524 nm λ_ex_- 420 nm	21.37%	2019	[30]
Durian shell	Tris base and deionized water	Hydrothermal carbonization	TEM- 6.5 nm	λ_em_- 414 nm λ_ex_- 340 nm	12.93%	2019	[31]
Lemon	Hydroxylamine	Hydrothermal	HRTEM- 2 nm	λ_em_- 430–470 nm λ_ex_- 360 nm	5%	2020	[32]
Pomegranate	Sodium hydroxide and polyethylene glycol	Microwave	HRTEM- 1 to 5 nm	λ_em_- 532 nm	-	2020	[33]
Watermelon peels				λ_em_- 515 nm			
*Rosa roxburghii* fruits	Water	Hydrothermal	TEM- 2.5 nm	λ_em_- 450 nm λ_ex_- 360 nm	24.8%	2020	[34]
Citrus fruit peels	Deionized water	Sand bath	TEM- 4.6 nm	λ_em_- 510 nm λ_ex_- 420 nm	-	2021	[35]
Banana peel	Deionized water	Hydrothermal	TEM- 5 nm	λ_ex_- 355 nm	20%	2021	[36]
*Elaeagnus angustifolia*	Ultrapure water	Hydrothermal	TEM- <10 nm	λ_em_- 410 nm λ_ex_- 330 nm	16.8%	2021	[37]
Kiwi (Actinidia deliciosa) fruit peels	-	Hydrothermal carbonization	TEM- 5.6 nm	λ_em_- 432 nm λ_ex_- 360 nm	14%	2021	[1]
	Ammonium hydroxide		TEM- 5.1 nm		19%		
Canon ball fruit	Distilled water	Hydrothermal	TEM- <15 nm	λ_em_- ~500 nm λ_ex_- 380 nm	7.24%	2022	[38]
Indian *Bael patra* -hard shell -pulp -pulp and gum	-	Hydrothermal carbonization	TEM- 3 nm TEM- 6 nm TEM- 8 nm		59.39% 59.07% 55.25%	2022	[39]
*Morus nigra* (black mulberry)	Deionized water	Hydrothermal	TEM- 4.5 nm	λ_em_- 427 nm λ_ex_- 360 nm	24%	2022	[40]
*Jatropha*	Distilled water	Hydrothermal	TEM- 3.2 nm	λ_em_- 462 nm λ_ex_- 370 nm	13.7%	2022	[41]

**Table 2:** Summary of the synthesis of CDs from vegetables.

Precursor		Technique	Properties			Year	Reference
Carbon Source	Passivation/Solvent		Particle Size	Fluorescence	Quantum Yield		
Celery leaves	Glutathione and double distilled water	Hydrothermal	TEM- 2.08 nm	λ_em_- 415 nm λ_ex_- 340 nm	53%	2013	[42]
Sweet pepper	Water	Carbonization	TEM- 4.6 nm	λ_em_- 450 nm λ_ex_- 360 nm	19.3%	2013	[43]
Lemon grass	Water	Hydrothermal	-	λ_em_- 440 nm λ_ex_- 320 nm	23.3%	2016	[44]
Tomato juice	-	Hydrothermal	HRTEM- 3 nm DLS- 3 nm	λ_em_- 440 nm λ_ex_- 367 nm	13.9%	2016	[45]
Carrot juice	-	Hydrothermal	TEM- 5.5 nm	λ_em_- 442–565 nm λ_ex_- 360–520 nm	5.16%	2017	[46]
Rose-heart radish	Ultrapure water	Hydrothermal	TEM- 3.6 nm	λ_em_- 420 nm λ_ex_- 330 nm	13.6%	2017	[47]
Turmeric	Ethylenediamine	Hydrothermal	TEM- 20 nm	-	20%	2018	[24]
Cinnamon	Ultrapure water	Hydrothermal	TEM- 3.4 nm	λ_em_- 465 nm λ_ex_- 370 nm	35.7%	2018	[48]
Red chili			TEM- 3.1 nm	λ_em_- 477 nm λ_ex_- 380 nm	26.8%		
Turmeric			TEM- 4.3 nm	λ_em_- 460 nm λ_ex_- 370 nm	38.3%		
Black pepper			TEM- 3.5 nm	λ_em_- 489 nm λ_ex_- 390 nm	43.6%		
Hongcaitai	Ultrapure water	Hydrothermal	TEM- 1.9 nm	λ_em_- 410 nm λ_ex_- 330 nm	21.0%	2018	[49]
Cauliflower	-	Hydrothermal	AFM- 4 nm DLS- 1.54 nm	λ_em_- 380 nm λ_ex_- 325 nm	43%	2019	[50]
Kelp	Ethylenediamine	Microwave irradiation	TEM- 3.7 nm	λ_em_- 450 nm λ_ex_- 370 nm	23.5%	2019	[51]
Tomato	Sulfuric acid	Chemical oxidation	HRTEM- 5–10 nm	λ_em_- 450 λ_ex_- 360 nm	12.70%	2019	[52]
	Phosphoric acid			λ_em_- 520 λ_ex_- 420 nm	4.21%		
	Phosphoric acid			λ_em_- 560 nm λ_ex_- 460 nm	2.76%		
Crown daisy leaf waste	Ultrapure water and urea	Hydrothermal	TEM- 5–10 nm	λ_em_- 380 nm λ_ex_- 300 nm	-	2019	[53]
Cabbage	Anhydrous ethanol	Solvothermal	TEM- 3.4 nm	λ_em_- 500 nm and 678 nm λ_ex_- 410 nm	12.4%	2020	[54]
Cherry tomatoes	-	Hydrothermal	TEM- 7 nm	λ_em_- 430 nm λ_ex_- 340 nm	9.7%	2020	[55]
Tomato	Hydroxylamine	Hydrothermal	HRTEM- 3 nm	λ_em_- 430–470 nm λ_ex_- 360 nm	3.38%	2020	[32]
Scallion leaves	Water	Hydrothermal	TEM- 3.5 nm	λ_em_- 418 nm λ_ex_- 320 nm	3.2%	2020	[56]
Tomato	-	Hydrothermal	HRTEM- 9 nm	λ_em_- 430 nm λ_ex_- 344 nm	1.24%	2021	[57]
Red beet	Water	Hydrothermal	TEM- 4.66 nm	λ_em_- 438 nm λ_ex_- 350 nm	8.17%	2022	[58]

**Table 3:** Summary of the synthesis of CDs from flowers.

Precursor		Technique	Properties			Year	Reference
Carbon Source	Passivation/Solvent		Particle Size	Fluorescence	Quantum Yield		
*Selenicereus grandiflorus*	-	Boiling	HRTEM- 2.5 nm	λ_em_- 440 and 365 nm	3.8%	2019	[59]
Water hyacinth	Phosphoric acid	Carbonization	SEM- ≤10 nm DLS- ≤10 nm TEM- 5.22 nm	λ_em_- 370 nm λ_ex_- 300 nm	17.02%	2019	[60]
*Osmanthus fragrans*	Ultrapure water	Hydrothermal	TEM- 2.23 nm	λ_em_- 410 nm λ_ex_- 340 nm	18.53%	2019	[61]
Rose flowers: Blue Red Yellow	Water	Hydrothermal	TEM- 37 nm TEM- 39 nm TEM- 33 nm	λ_ex_- 335 nm λ_ex_- 330 nm λ_ex_- 340 nm	46% 44% 48%	2020	[62]
	Ethanol		TEM- 30 nm TEM- 27 nm TEM- 26 nm	λ_ex_- 420 nm λ_ex_- 410 nm λ_ex_- 425 nm	43% 46% 47%		
*Tagetes erecta*	Deionized water	Solvo(hydro)-thermal carbonization	FESEM- 3.41 nm	λ_em_- 495 nm λ_ex_- 420 nm	63.7%	2021	[63]

**Table 4:** Summary of the synthesis of CDs from leaves, seeds, and stems.

Precursor		Technique	Properties			Year	Reference
Carbon Source	Passivation/Solvent		Particle Size	Fluorescence	Quantum Yield		
Lotus root	-	Microwave	TEM- 9.41 nm	λ_em_- 435 nm λ_ex_- 360 nm	19.0%	2016	[64]
*Ocimum sanctum* leaves	Distilled water	Hydrothermal	TEM- 2.23 nm	λ_em_- 410 nm λ_ex_- 340 nm	9.3%	2017	[65]
*Acacia concinna* seeds	Methanol	Microwave treatment	HRTEM- 2.5 nm	λ_em_- 468 nm λ_ex_- 390 nm	10.20%	2018	[66]
	Acetonitrile				7.20%		
	Acetone				7.85%		
Bamboo leaves	Sodium hydroxide and sodium hypochlorite	Pyrolysis	AFM- 2 nm	λ_em_- 425–475 nm	-	2018	[67]
Gingko leaves	-	Pyrolysis	TEM- 4.11 nm	λ_em_- 427 nm λ_ex_- 360 nm	21.7%	2018	[68]
*Gynostemma*	-	Calcination	TEM- 2.5 nm	λ_em_- 400 nm λ_ex_- 320 nm	5.7%	2019	[69]
Fennel seeds (*Foeniculum vulgare*)	-	Pyrolysis	TEM- 3.9 nm	λ_em_- 417 nm λ_ex_- 240 nm	9.5%	2019	[70]
Bamboo leaves	-	Calcination	TEM- 11 nm	λ_em_- 419 nm λ_ex_- 313 nm	5.18%	2020	[71]
Betel leaves	Ammonia	Hydrothermal	HRTEM- less 10 nm PSA-3.7 nm	λ_em_- 402 nm λ_ex_- 320 nm	4.21%	2021	[72]
*Calotropis procera* leaves	Deionized water	Hydrothermal carbonization	FETEM- 4.3 nm	λ_em_- 416 nm λ_ex_- 340 nm	71.95%	2021	[73]
*Elettaria cardamomum* leaves	Distilled water	Ultrasonication	-	λ_em_- 520 and 850 nm λ_ex_- 514 nm	-	2021	[74]
*Pearl millet* seeds	Double distilled water	Thermal treatment	HRTEM- 4–5 nm	λ_em_- 415 nm λ_ex_- 250 nm	52%	2021	[75]
Cornus walteri leaves	Maleic anhydride, hydrogen peroxide and water	Hydrothermal	TEM- 3.53 nm	λ_em_- 550 nm λ_ex_- 420 nm	18.34%	2022	[76]
Tea leaves	Urea and ultrapure water	Hydrothermal	TEM- 2.32 nm	λ_em_- 455 nm λ_ex_- 360 nm	-	2022	[77]
Kentucky bluegrass	Ethylenediamine	Hydrothermal	TEM- 9 nm	λ_em_- 370–470 nm λ_ex_- 280–400 nm	7%	2022	[78]

**Table 5:** Summary of the synthesis of CDs from crop residues.

Precursor		Technique	Properties			Year	Reference
Carbon Source	Passivation/Solvent		Particle Size	Fluorescence	Quantum Yield		
Sago waste	-	Thermal pyrolysis	SEM- 6–17 nm	λ_em_- 390 nm λ_ex_- 315 nm	-	2014	[79]
Palm kernel shell	Diethylene glycol	Microwave irradiation	TEM- 6.6 to 7 nm	λ_em_- 438–459 nm λ_ex_- 370 nm	44.0%	2020	[80]
Palm kernel shell	Ultrapure water and ethylenediamine	Hydrothermal	TEM-2 nm	λ_em_- 430–450 nm λ_ex_- 350–400 nm	13.7%	2021	[81]
	Ethanol and L-phenylalanine				8.6%		
Wheat straw	Deionized water	Hydrothermal	TEM- 2.1 nm DLS- 5.7 nm	λ_em_- 470 nm λ_ex_- 380 nm	7.5%	2021	[82]

**Table 6:** Summary of the synthesis of CDs from fungi/bacteria species.

Precursor		Technique	Properties			Year	Reference
Carbon Source	Passivation/Solvent		Particle Size	Fluorescence	Quantum Yield		
Algal blooms	Phosphoric acid	Microwave	TEM- 8.5 nm	λ_em_- 438 nm λ_ex_- 360 nm	13%	2016	[83]
Yogurt	Hydrochloric acid	Pyrolysis	TEM- 3.5 nm	λ_em_- 420 nm λ_ex_- 320 nm	2.4%	2018	[84]
Enokitake mushroom	Sulfuric acid	Hydrothermal	TEM- 4 nm	λ_em_- 470 nm λ_ex_- 360 nm	11%	2018	[85]
	Sulfuric acid and tetraethylenepentamine				39%		
Microalgae biochar	Potassium permanganate	Oxidizing agent and autoclave	AFM- 68 nm	λ_ex_- 398 nm λ_ex_- 280 nm	-	2019	[86]
Mushroom	Ultrapure water	Hydrothermal	TEM- 5.8 nm	λ_em_- 440 nm λ_ex_- 360 nm	11.5%	2020	[87]
Agarose waste	-	Thermal treatment	HRTEM- 2–10 nm	λ_em_- 420 nm λ_ex_- 300 nm	62%	2021	[88]
*Shewanella oneidensis*	Luria-Bertani	Hydrothermal	-	λ_em_- 410 nm λ_ex_- 320 nm	7%	2022	[89]

**Table 7:** Summary of the synthesis of CDs from waste products.

Precursor		Technique	Properties			Year	Reference
Carbon Source	Passivation/Solvent		Particle Size	Fluorescence	Quantum Yield		
Waste frying oil	Sulfuric acid	Carbonization	HRTEM- 2.6 nm	λ_em_- 378 nm λ_ex_- 300 nm	3.66%	2014	[90]
Nannochloropsis biocrude oil	Sulfuric acid	Hydrothermal liquefaction	TEM- 4 nm	λ_em_- 280–560 nm λ_ex_- 400–550 nm	13.71%	2017	[91]
Polystyrene	Ethylenediamine	Solvothermal	TEM- 4 nm	λ_em_- 456 nm λ_ex_- 380 nm	~20%	2017	[92]
Assam CTC tea	Acetic acid	Carbonization	TEM- <10 nm	λ_ex_- 380–500 nm	-	2017	[93]
Expired milk	Water	Subcritical water	TEM- <5 nm	λ_em_- 440 nm λ_ex_- 360 nm	8.64%	2018	[94]
Long flame coal powder	Deionized water	Ozone oxidation	DLS- 4.2 nm	λ_em_- 530 nm λ_ex_- 470 nm	8.4%	2018	[95]
Paper waste	Sodium hydroxide and distilled water	Hydrothermal	TEM- 2–4 nm	λ_em_- 420 nm λ_ex_- 320 nm	20%	2018	[96]
Waste polyolefin	Sulfuric acid and nitric acid	Ultrasonic-assisted chemical oxidation	TEM- 2.5 nm	λ_em_- 540 nm λ_ex_- 490 nm	4.84%	2018	[97]
Polypropylene plastic waste	Ethanol	Heating process	TEM- <20 nm	λ_em_- 410–465 nm λ_ex_- 365 nm	-	2018	[98]
Bike soot	Deionized water	Hydrothermal	TEM- 4.2 nm	λ_em_- 396 nm λ_ex_- 240 nm	~5.63%	2018	[99]
	Nitric acid		TEM- 5.6 nm	λ_em_- λ_ex_-	3.25%		
	Phosphoric acid			λ_em_- 560 nm λ_ex_- 460 nm	2.76%		
Coke powder	Hydrogen peroxide	Chemical oxidation	TEM- 6.5 nm	λ_em_- 410 nm λ_ex_- 330 nm	9.2%	2019	[100]
Waste plastic bottles	Hydrogen peroxide	Air oxidation and hydrothermal	TEM- 3–10 nm	λ_em_- 434 nm λ_ex_- 340 nm	5.2%	2019	[101]
Waste tea powder	Nitric acid	Chemical oxidation	TEM- 3.2 nm	λ_em_- 430 nm λ_ex_- 310 nm	2.47%	2019	[102]
Waste tea powder	-	Carbonization	TEM- 5 nm	λ_em_- 415 nm λ_ex_- 315 nm	4.76%	2019	[103]
Waste green tea powder	Deionized water and manganese chloride	Hydrothermal	TEM- 5 nm	λ_em_- 410–440 nm λ_ex_- 360 nm	12%	2019	[104]
Tieguanyin Tea leaves	Acetic acid	Hydrothermal	TEM- 7–9 nm	λ_em_- blue λ_ex_- 325 nm	-	2019	[105]
Peanut shell				λ_em_- blue λ_ex_- 335 nm			
Kerosene soot	Nitric acid	Oxidative acid treatment	HRTEM- 5 nm	λ_em_- 510 nm λ_ex_- 300–360 nm	~3%	2019	[106]
Paper waste	Deionized water	Hydro-/solvothermal	TEM- 2.6 nm	λ_em_- blue λ_ex_- 360 nm	12%	2020	[107]
	Ethanol		TEM- 4.0 nm	λ_em_- 435 nm (cyan) λ_ex_- 360 nm	27%		
	2-propanol		TEM- 4.4 nm	λ_em_- 435 nm (cyan) λ_ex_- 360 nm	10%		
Polybags	-	Hydrothermal carbonization	HRTEM- 5–10 nm	λ_em_- 420–425 nm λ_ex_- 310 nm	62%	2021	[108]
Cups				λ_em_- 420–425 nm λ_ex_- 310 nm	65%		
Bottles				λ_em_- 420–425 nm λ_ex_- 310 nm	64%		
Polymeric waste	-	Hydrothermal	TEM- 3 nm	λ_em_- 400 nm λ_ex_- 310 nm	-	2021	[109]
	4,7,10-trioxa-1,13-tridecanediamine			λ_em_- 440 nm λ_ex_- 365 nm			
Heavy oil	-	Hydrothermal	-	-	-	2021	[110]
Light deasphalted oil (LDAO)			-	-	~64%		
Heavy deasphalted oil (HDAO)			-	-	23.5%		
Asphalt			TEM- 2.39 nm	λ_em_- 610 nm λ_ex_- 475 nm	11.5%		
			TEM- 1.77 nm	λ_em_- 560 nm λ_ex_- 420 nm	17.7%		
			TEM- 1.21 nm	λ_em_- 510 nm λ_ex_- 410 nm	28.3%		
			TEM- 1.18 nm	λ_em_- 440 nm λ_ex_- 350 nm	64%		
Waste tobacco leaves	Ethylenediamine and ultrapure water	Hydrothermal	TEM- 6.30 nm	λ_em_- 430 nm λ_ex_- 360 nm	13.7%	2022	[111]

**Table 11:** Summary of the developed optical sensors for phenol detection.

Phenolic Compound	Material	Optical Sensor	Range of Detection	Limit of Detection	Linear Correlation Coefficient	Year	Reference
2-4-6-Trinitrophenol	Amine-capped CDs	Fluorescent	0–50 × 10^−5^ M	0.9996 μM	-	2013	[114]
2-4-6-Trinitrophenol	Tb-CDs	Fluorescent	500 nM–100 μM	200 nM	0.991	2013	[112]
4-Nitrophenol	CDs	Fluorescent	0.1–50 µM	28 nM	-	2014	[115]
Tannic acid	PEGA-CDs	Fluorescent	0.05–0.6 μM	0.01 μM	-	2015	[204]
2-4-6-Trinitrophenol	N-CQDs	Fluorescent	0.27–34.1 µM	50 nM	0.992	2016	[134]
2-4-6-Trinitrophenol	CDs	Fluorescent	0.1–26.5 µM	51 nM	0.995	2017	[136]
2-4-6-Trinitrophenol	CDs	Fluorescent	-	0.127 µM	-	2018	[150]
Phenol	CDs	Fluorescent	0–50 µM	0.076 µM	0.998	2018	[95]
Tannic acid	N-CDs	Fluorescent	0.4–9.0 µM	0.12 µM	0.9990	2019	[167]
4-Nitrophenol	CDs@MIPs	Fluorescent	0–0.03594 mM	35 nM	-	2019	[165]
p-Nitrophenol	Cu-doped carbon dots	Fluorescent	0.5–50 μM	0.08 μM	0.998	2019	[218]
2-4-6-Trinitrophenol	CDs	Fluorescent	0–305.54 μM	0.023 μM	-	2020	[170]
o-Nitrophenol	CQDs	Fluorescent	0.08–40 µM	15.2 nM	0.999	2020	[34]
4-Nitrophenol	N,CDs	Fluorescent	0.25–125 μM	0.05 μM	0.9919	2020	[222]
2-4-6-Trinitrophenol	N@CDs	Fluorescent	1–75 μM	2.45 μM	0.994	2020	[221]
Trinitrophenol	wsNP-CDs	Fluorescent	100–300 μM	23 μM	0.9861	2020	[243]
Tannic acid	Nitrogen-doped CDs	Chemiluminescence	0.2–10 μM	39.3 nM	0.9971	2020	[173]
Chlorogenic acid	N,S-CDs	Fluorescent	0.9314–83.82 μM	0.3387 μM	0.9970	2021	[184]
4-Nitrophenol	CDs@PDA	Fluorescent	2–34 μM	7.29 μM 3.44 μM	0.992 0.993	2021	[226]
2-4-6-Trinitrophenol	N-CDs	Fluorescent	0.3–3.3 μM	0.11 μM	0.9923	2021	[72]
p-Nitrophenol	G-CDs	Fluorescent	0–50 μM	0.0175 μM	0.9951	2022	[76]

Where Tb-CDs: terbium doped carbon dots, PEGA-CDs: polyethyleneglycol bis(3-aminopropyl)-carbon dots, CDs@MIPs: carbon dots fabricated with molecularly imprinted polymers, N@CDs: nitrogen doped carbon dots, wsNP-CDs: water soluble nitrogen and phosphorous doped carbon dots, CDs@PDA: polydopamine encapsulated carbon dots, G-CDs: green-emitting carbon dots.

**Table 12:** Summary of the developed optical sensors for pesticides detection.

Pesticide	Material	Optical Sensor	Range of Detection	Limit of Detection	Linear Correlation Coefficient	Year	Reference
Methyl parathion	Tyr-CDs	Fluorescent	1.0 × 10^−10^–1.0 × 10^−4^ M	4.8 × 10^−11^ M	0.997	2015	[121]
Paraoxon-ethyl	CDs	Fluorescent	0–5.80 mM	0.22 µM	0.9974	2016	[137]
Dichlorvos, malathion, ethion	CDs/Cu(II)/AChE/ATChCl	Fluorescent	6 nM–0.6 nM 6 nM–0.8 nM 8 nM–0.8 nM	3.8 nM 3.4 nM 4.2 nM	0.998 0.996 0.997	2016	[245]
Carbaryl	N,S co-doped CQDS	Photoluminescent	0.00003131–3.131 µM	0.02485 µM	-	2016	[209]
Paraoxon	CQDs	Fluorescent	0.1817–181.7 nM	0.1817 nM	0.994	2017	[211]
Paraoxon	BChE-ATCh-MnO_2_-CDs	Fluorescent	0.1817–18.17 nM	0.05451 nM	0.9941	2017	[155]
Chlorpyrifos	Fe-modified CDs	Fluorescent	0.028523–2.8523 µM	0.008557 µM	-	2017	[96]
Methyl parathion	N-doped CDs-MPH	Fluorescent	2.38–73.78 µM	0.338 µM	0.9934	2017	[138]
Paraoxon	CDs	Fluorescent	0–1.817 µM	0.00145 µM	0.993	2018	[154]
Atrazine	N-CQDs	Fluorescent	0–1.0 nM	3 pM	0.9812	2018	[213]
Atrazine; chlorpyrifos; imidacloprid; lindane; tetradifon	CDs	Fluorescent	-	0.12 µM; 0.029 µM; 0.013 µM; 0.14 µM; 0.04 µM	-	2019	[246]
Pretilachlor	CDs	Fluorescent	5.7 μM–61.5 μM	2.9 µM	0.9847	2019	[60]
Diazinon	CDs	Fluorescent	0.8214 nM–16.43 μM	0.8214 nM	-	2020	[50]
Glyphosate			1.4787 nM–29.574 μM	0.01183 μM			
Amicarbazone			1.036 nM–20.72 μM	0.002072 μM			
Methyl-paraoxon	B,N-CDs	Fluorescent	0.1–15 μM	0.1 μM	0.9967	2020	[180]
Diazinon	CDs	Fluorescent	0.02–10 μM	0.01 μM	0.9727	2020	[62]
Trifluralin	Ca-modified CDs	Fluorescent	-	7.89 µM	0.96	2020	[220]
Quinalphos	OPCD@UiO-66-NH_2_	Fluorescent	0–16 μM	0.3 nM	0.992	2021	[247]
Chlorpyrifos	TEF-CDs	Fluorescent	0.05–100.0 μM	0.00599 μM	0.9959	2021	[63]
Quinalphos			0.01–50.0 μM	0.0057 μM	0.9965		
Pyrimethanil	CDs	Fluorescent	0.5–75 μM	14 nM	0.9907	2021	[182]
Thiophanate methyl	SCDs/Hg^2+^	Fluorescent	0.05–2.0 μM	7.6 nM	0.9998	2021	[183]
			2.0–5.0 μM		0.9983		
Isoprothiolane	CDs	Fluorescent	1 mM-0.05 μM	11.58 nM	0.9921	2021	[73]
λ-Cyhalothrin	CD-functionalized core-shell nanopsheres	Ratiometric fluorescent	3.045–456.8 nM	0.146 nM	0.988	2022	[230]
Chlorpyrifos	J-CQDs	Fluorescent	57.05–513.4 nM	7.701 nM	0.993	2022	[41]

Where Tyr-CDs: L- tyrosine methyl ester functionalized carbon dots, CDs/Cu(II)/AChE/ATChCl: carbon dots/copper ion/acetyl cholinesterase/acetylthiocholine, BChE-ATCh-MnO_2_-CDs: butyrylcholinesterase- acetylthiocholine- manganese dioxide- carbon dots, N-doped CDs-MPH: nitrogen-doped carbon dots and methyl parathion hydrolase, B,N-CDs: boron and nitrogen-doped carbon dots, Ca-modified CDs: calcium-modified carbon dots, OPCD@UiO-66-NH_2_: carbon dots derived from orthophenylenediamine incorporated UiO-66-NH_2_, TEF-CDs: carbon dots from *Tagetes erecta* flower, SCDs/Hg^2+^: sulfur-doped carbon dots/mercury ions, J-CQDs: Jatropha-carbon quantum dots.

**Table 13:** Summary of the developed optical sensors for explosive compound detection.

Explosive Compound	Material	Optical Sensor	Range of Detection	Limit of Detection	Linear Correlation Coefficient	Year	Reference
2,4,6-Trinitrotoluene	N-rich CNDs	Fluorescent	10 nM–1.5 μM	1 nM	-	2015	[120]
2,4-Dinitrotoluene	Amine-functionalized CDs	Fluorescent	1 mM–50 mM	1 mM	-	2016	[132]
Trinitrotoluene	M-MIPs@CDs	Fluorescent	-	17 nM	-	2016	[131]
4-Chloro-2,6-dinitroaniline	CQDs@PAMAM-NH_2_	Fluorescent	1.0 × 10^−5^–6.0 × 10^−5^ M	2 μM	0.994	2016	[208]
2,4,6-Trinitrotoluene	Ethylenediamine-modified CDs	Fluorescent	-	0.213 µM	0.997	2017	[139]
Trinitrotoluene	Nitrogen-doped CQD	Fluorescent	4.4 nM–26.4 µM	0.03258 µM	0.9993	2018	[214]
Trinitrotoluene	CDs capped with EDA	Fluorescent	44.03–220.14 nM	57.24 nM	0.95457	2018	[99]
	CDs (in the presence of nitric acid) capped with\EDA		44.03–220.14 nM	48.43 nM	0.9752		
			88.06–264.17 nM	21.88 nM	0.99602		
2,4,6-Trinitrotoluene	CDs and Fe@SiO_2_-NH_2_	Chemosensor	44.03–8806 nM	9.466 nM	-	2021	[248]
2,4,6-Trinitrotoluene	PEI-CQDs	Fluorescent	0–38.17 µM	0.4094 µM	0.9979	2022	[196]

Where N-rich CNDs: nitrogen-rich carbon nanodots, M-MIPs@CDs: mesoporous structured molecularly imprinted polymers capped carbon dots, CQDs@PAMAM-NH_2_: carbon quantum dots functionalized with amine groups by PAMAM dendrimer, EDA: ethylenediamine, CDs and Fe@SiO_2_-NH_2_: carbon dots and the magnetism of amino-functionalized magnetic core-shell nanomaterial, PEI-CQDs: polythylenimine capped carbon quantum dots.

## Data Availability

Not applicable.
